# Gene expression and promoter methylation of angiogenic and lymphangiogenic factors as prognostic markers in melanoma

**DOI:** 10.1002/1878-0261.12501

**Published:** 2019-05-25

**Authors:** Ana Carolina Monteiro, Julienne K. Muenzner, Fernando Andrade, Flávia Eichemberger Rius, Christian Ostalecki, Carol I. Geppert, Abbas Agaimy, Arndt Hartmann, André Fujita, Regine Schneider‐Stock, Miriam Galvonas Jasiulionis

**Affiliations:** ^1^ Department of Pharmacology Escola Paulista de Medicina Universidade Federal de São Paulo Brazil; ^2^ Department of Experimental Tumor Pathology Institute of Pathology Friedrich‐Alexander‐Universität Erlangen‐Nürnberg (FAU) Germany; ^3^ Department of Computer Science Institute of Mathematics and Statistics Universidade de São Paulo Brazil; ^4^ Department of Dermatology Friedrich‐Alexander Universität Erlangen‐Nürnberg (FAU) Universitätsklinikum Erlangen Germany; ^5^ Institute of Pathology Friedrich‐Alexander‐Universität Erlangen‐Nürnberg (FAU) Germany

**Keywords:** angiogenesis, biomarkers, DNA methylation, melanoma, prognosis

## Abstract

The high mortality rate of melanoma is broadly associated with its metastatic potential. Tumor cell dissemination is strictly dependent on vascularization; therefore, angiogenesis and lymphangiogenesis play an essential role in metastasis. Hence, a better understanding of the players of tumor vascularization and establishing them as new molecular biomarkers might help to overcome the poor prognosis of melanoma patients. Here, we further characterized a linear murine model of melanoma progression and showed that the aggressiveness of melanoma cells is closely associated with high expression of angiogenic factors, such as *Vegfc*,* Angpt2, and Six1,* and that blockade of the vascular endothelial growth factor pathway by the inhibitor axitinib abrogates their tumorigenic potential *in vitro* and in the *in vivo* chicken chorioallantoic membrane assay. Furthermore, analysis of The Cancer Genome Atlas data revealed that the expression of the angiogenic factor *ANGPT2* (*P*‐value = 0.044) and the lymphangiogenic receptor *VEGFR‐3* (*P*‐value = 0.002) were independent prognostic factors of overall survival in melanoma patients. Enhanced reduced representation bisulfite sequencing‐based methylome profiling revealed for the first time a link between abnormal *VEGFC*,* ANGPT2,* and *SIX1* gene expression and promoter hypomethylation in melanoma cells. In patients, *VEGFC* (*P*‐value = 0.031), *ANGPT2* (*P*‐value < 0.001), and *SIX1* (*P*‐value = 0.009) promoter hypomethylation were independent prognostic factors of shorter overall survival. Hence, our data suggest that these angio‐ and lymphangiogenesis factors are potential biomarkers of melanoma prognosis. Moreover, these findings strongly support the applicability of our melanoma progression model to unravel new biomarkers for this aggressive human disease.

**Abbreviations**
ANGPT: angiopoietin
CAM: chicken chorioallantoic membrane
FGF: fibroblast growth factor
IRS: immunoreactive score
PDGF: placental growth factor
TCGA: The Cancer Genome Atlas
VEGFR: vascular endothelial growth factor receptor
VEGF: vascular endothelial growth factor

## 1 Introduction

Melanoma mortality rate, one of the highest among all human cancers, is broadly associated with its metastatic potential (Tímár *et al*., 2016). Although the complete resection of localized melanomas is curative in nearly all cases (Shain and Bastian, 2016), the survival rate for patients identified with metastatic melanoma is only 6–11 months (Clark *et al*., 2018; Fruehauf *et al*., 2011).

Metastasis is a complex process, in which a sufficient blood supply is critical for dissemination and subsequent tumor growth. Melanoma cells can spread through hematogenous and lymphatic routes; therefore, the formation of new blood and lymphatic vessels via angiogenesis and lymphangiogenesis, respectively, is crucial (Adler *et al*., 2017). Indeed, high vascularization has been associated with melanoma progression (Chung and Mahalingam, 2014; Tímár *et al*., 2016).

Melanoma cells produce several proangiogenic factors, including vascular endothelial growth factor (VEGF), fibroblast growth factor (FGF), placental growth factor (PDGF), and angiopoietin (ANGPT), which are involved in tumor vascularization (Matsumoto and Claesson‐Welsh, 2001). One of the major players is the VEGF family, which is comprised of the glycoproteins VEGFA, VEGFB, VEGFC, VEGFD, and PDGF, and their tyrosine kinase receptors vascular endothelial growth factor receptor (VEGFR)‐1 (FLT1), VEGFR‐2 (KDR), VEGFR‐3 (FLT4), and co‐receptor NRP. The VEGFA signaling pathway is classically known to induce angiogenesis by the activation of VEGFR‐2, while VEGFR‐3, activated by VEGFC and VEGFD, is specifically related to lymphangiogenesis (Matsumoto and Claesson‐Welsh, 2001). Notably, cutaneous melanomas have a high metastatic potential to lymph nodes, which is associated with high expression of VEGFC (Adler *et al*., 2017; Boone *et al*., 2008).

As mentioned, diverse molecules are involved in vessel formation. VEGFs are associated with the early steps of blood vessels formation, while ANGPTs are critical regulators of vascular and lymphatic maturation and remodeling (Rigamonti *et al*., 2014; Thurston, 2003). ANGPT1 and ANGPT2 bind to the tyrosine kinase receptor TIE2; the former is considered an agonist and the latter primarily an antagonist of this receptor. ANGPT2 has been shown to be highly expressed in tumors, promoting endothelial disruption and facilitating tumor cell extravasation (Li *et al*., 2015).

Angiogenic factors have been shown to be useful in predicting cancer progression and aggressiveness in different malignancies and were proposed as tumor biomarkers (Cao *et al*., 2014; Martinelli *et al*., 2013). Studies with melanoma patients show conflicting results; while some have shown that VEGFA and VEGFC can predict shorter overall and disease‐free survival (Boone *et al*., 2008; Spiric *et al*., 2015; Tas *et al*., 2006), other studies failed to prove such a correlation (Bolander *et al*., 2007; Vihinen *et al*., 2007). Recently, it has been reported that the promoter regions of the major angiogenesis players contain extended CpG islands (Pirola *et al*., 2018). Thus, DNA methylation might be a potent regulatory mechanism of angiogenesis in melanoma.

Here, we show that the aggressiveness of murine melanoma cells is closely associated with high expression of angiogenic factors and that blockade of the VEGF pathway abrogates the tumorigenic potential of metastatic melanoma cells. Furthermore, the expression of the angiogenic factor *ANGPT2* and the receptor *VEGFR‐3* were significantly associated with overall survival of melanoma patients. The methylation status of *VEGFC, ANGPT2,* and SIX homeobox 1 (*SIX1*) promoters was also found to correlate with overall survival in melanoma patients. In the studied murine model, DNA methylation was identified as the mechanism regulating the abnormal expression of these genes.

## 2 Methods

### 2.1 Cell lines and drug treatment

Murine melanoma cell lines 4C11− (nonmetastatic) and 4C11+ (metastatic) were cultured in RPMI 1640 medium supplemented with 5% FBS and 1% penicillin (100U·mL^−1^) and streptomycin (100 μg·mL^−1^) at 37 °C in 5% CO_2_ humidified atmosphere. Cell culture reagents were purchased from PAN Biotech (Aidenbach, Germany). Axitinib (PZ0193; Sigma‐Aldrich, St. Louis, MO, USA), a selective inhibitor of VEGF receptors, and 5‐Aza‐2′‐deoxycytidine (5‐Aza‐CdR; Calbiochem, Merck, Darmstadt, Germany) were dissolved in DMSO (PAN Biotech) and stored at a final concentration of 10 mm at −20 °C. 4C11+ cells were treated with different concentrations (40 nm‐10 μm) of axitinib for MTT assay and with 1 μm for all other assays. All treatments were performed for 48 h. 4C11− cells were treated with 10 μm of 5‐Aza‐CdR for 72 h. As a control, cells were treated with the respective volume of DMSO. Final DMSO volume in the cell culture was lower than 0.01%.

### 2.2 *In vivo* chicken chorioallantoic membrane assay

The chicken chorioallantoic membrane (CAM) assay was performed as previously described (Muenzner *et al*., 2018). Fertilized specific pathogen‐free chicken eggs were obtained from Valo BioMedia (Osterholz‐Scharmbeck, Germany) and incubated at 37 °C with ~ 80% relative humidity. The first day of incubation was considered as embryonic day (EDD) 1. On EDD 8, the eggshell was opened at the more rounded pole of the egg and the exposed membrane residing below the air sac was removed with fine forceps revealing the CAM, and the window was re‐sealed with adhesive silk tape. On EDD 9, 4C11− and 4C11+ cells or pretreated 4C11+ cells (axitinib or vehicle) (1 × 10^6^ cells/egg) were applied on the CAM. Cells were prepared in a mixture of 50% RPMI medium and 50% Matrigel (Corning® Matrigel® Basement Membrane Matrix, 356237; Corning, Bedford, MA, USA), and the formed pellets were incubated for 1 h at 37 °C before being applied onto the CAM. Tumors and the adjacent CAM were dissected on EDD 12 or EDD 15. Tumor volume was measured (l × w × h × 0.526, where l indicates length, w indicates width, and h indicates height), and the tissue was fixed in 4% phosphate‐buffered formaldehyde before being embedded in paraffin for histopathologic observation. After the tumor and CAM had been removed, the embryo was immediately euthanized by decapitation.

### 2.3 Immunostaining

Immunohistochemistry (IHC) was performed to detect pH3 [phospho‐histone H3 (BC37), 1 : 200; Biocare Medical, Pacheco, CA, USA] in formalin‐fixed paraffin‐embedded (FFPE) tissue obtained from the CAM assay (*n* = 7); and VEGFR‐3 [(D6), 1 : 200; Santa Cruz, Dallas, TX, USA] and ANGPT2 [(F‐1), 1 : 100; Santa Cruz] in primary and metastatic FFPE human melanoma specimens (*n* = 5). The study methodologies conformed to the standards set by the Declaration of Helsinki. Briefly, sections (2–4 μm) were deparaffinized at 72 °C for 30 min, incubated in xylene, and rehydrated in EtOH. Antigen was retrieved by heating in a Tris/EDTA buffer at 120 °C for 5 min, and endogenous peroxidases and nonspecific binding sites were blocked with specific blocking solution. The slices were incubated with specific primary antibody and next with secondary horseradish peroxidase‐linked antibody. Positive immunoreactivity was detected using diaminobenzidine or AEC/H_2_O_2_, and nuclei were counterstained with hematoxylin and eosin (HE). Immunoreactive score (IRS) was assessed as described previously (Dumitru *et al*., 2013).

Hematoxylin and eosin‐stained tumor slices obtained from the CAM assay were scanned with Panoramic MIDI system (Camera type: CIS VCC‐FC60FR19CL, Objective name: Plan‐Apochromat, Objective magnification: 40×, Camera adapter magnification: 1×) (3DHISTECH, Budapest, Hungary), which generates a digital image with high quality, and evaluated with the caseviewer software (Version 2.0; 3DHISTECH).

Immunofluorescence images of cryosections of primary (*n* = 3) and metastatic (*n* = 3) melanoma were generated using the multi‐epitope ligand cartography technique (MELC). Sample preparations from tissue, data generation, and analysis were performed as described previously (Ostalecki *et al*., 2017). The following antibodies were used: ANGPT2‐Alexa Fluor 488 [(MM0020‐1F29), 1 : 40; Novus Biologicals, Centennial, CO, USA], VEGFR‐3‐PE [(9D9F9), 1 : 20; BioLegend, San Diego, CA, USA], and Collagen IV‐FITC [(5K134), 1 : 200; Biomol, Hamburg, Germany).

### 2.4 Total RNA isolation

Total RNA from 4C11− and 4C11+ melanoma cells was prepared using the miRNeasy Mini kit (Qiagen, Hilden, Germany), and DNA digestion was performed with RNase‐Free DNase Set (Qiagen) according to the manufacturer's protocol.

### 2.5 NanoString gene expression analysis

mRNA signature of 4C11− and 4C11+ cancer cells was profiled with nCounter PanCancer Mouse Pathways Panel (NanoString Technologies, Seattle, WA, USA). As recommended by the manufacturer, 100 ng of total RNA was used as input for sample preparation. Samples and specific probes were hybridized overnight at 65 °C, automatically processed in the Prep Station, and transferred to the Digital Analyzer for data collection with a high‐density scan (600 fields of view). NanoString nSolver software was used for normalization and pairwise comparisons. mRNA data were normalized with a set of 20 predetermined housekeeping genes. Based on background detection, the minimal threshold for detection was considered as 50 counts.

### 2.6 RT‐qPCR

Following the manufacturer's instructions, 1 μg of purified RNA was reverse transcribed to cDNA using miScript® II RT kit (Qiagen) with HiFlex Buffer. Real‐time PCR was performed with QuantiTect SYBR® Green PCR Kit (Qiagen) using 40 ng of cDNA and specific primers designed with NCBI Primer‐Blast software (Table S1). The PCR amplification conditions were as follows: 95 °C for 15 min, 40 cycles of 94 °C for 15 s, 60 °C for 30 s, and 70 °C for 30 s. Beta‐actin was used as a housekeeping control, and data were analyzed with the comparative 2^−ΔΔCT^ method. PCR reactions were performed using the Bio‐Rad CFX96 Real‐Time PCR Detection System.

### 2.7 Invasion assay

Membranes of Transwell® inserts (Corning) were precoated with Matrigel Growth Factor Reduced (356231; Corning) diluted in serum‐free medium in a 1 : 4 proportion. After Matrigel had jellified, 2 × 10^5^ previously serum‐starved (24 h) cells were seeded in the upper chamber in serum‐free medium. RPMI containing 10% FBS was used as a chemoattractant in the lower chamber. Cells were allowed to invade for 48 h at 37 °C and 5% CO_2_. After this period, membranes were fixed with 4% formaldehyde and stained with crystal violet. Images of invading cells were obtained in a bright‐field microscope (Leica DMi1; Leica Microsystems, Wetzlar, Germany).

### 2.8 Tube formation assay

A 96‐well plate was precooled, and 40 μL of Matrigel® (356237; Corning) was added in each well. The plate was incubated at 37 °C for at least 30 min to allow the Matrigel® to form a gel‐like structure before 1–6 × 10^4^ cells were seeded on top of the Matrigel® in 100 μL of complete medium. Tube formation was assessed after 16–18 h under a bright‐field microscopy (Leica DMi1).

### 2.9 MTT assay

Four thousand 4C11+ cells were seeded in the wells of a 96‐well plate and allowed to attach for 24 h. Following this, cells were treated with different concentrations of axitinib (Sigma‐Aldrich, Darmstadt, Germany) for 48 h and cell viability was analyzed by 3‐(4,5‐dimethylthiazol‐2‐yl)‐2,5‐diphenyltetrazolium bromide (MTT) reduction. For this, cells were incubated with MTT (Sigma‐Aldrich, Germany; 0.5 mg·mL^−1^) for 2 h at 37 °C, and the produced purple formazan was solubilized with DMSO and quantified at 595 nm with a reference filter of 620 nm in a multilabel plate reader (VictorTM X3 2030 Multilabel Plate Reader; Perkin Elmer, Waltham, MA, USA).

### 2.10 Wound healing assay

Cells were seeded in a 12‐well plate, were led to adhere for 24 h, and then treated with axitinib or vehicle alone for 48 h. At this point, the monolayer confluence was reached and a straight wound was created with a sterile 100 μL pipette tip in the center of each well and cell debris were eliminated by washing with PBS. Cells were incubated with FBS‐free medium containing 2 ng·μL^−1^ of mitomycin C (Sigma‐Aldrich), a mitosis inhibitor, and cell migration was monitored for 48 h by bright‐field microscopy (Nikon, Tokyo, Japan). The cell‐free area was measured with imagej software (National Institutes of Health, Bethesda, MD, USA), and relative cell migration was quantified by the equation: Migration % = [1 − (cell‐free area at *t*_24_/cell‐free area at *t*_0_) × 100].

### 2.11 Cell cycle analysis

For cell cycle analysis, after the appointed treatment, adherent and occasional floating cells in the supernatant were collected, washed in PBS, and fixed overnight with ice‐cold 70% EtOH. Next, cells were incubated with staining solution containing 50 μg·mL^−1^ propidium iodide and 0.5 mg·mL^−1^ RNase for 30 min in the dark. DNA content was determined in FACS Canto® II flow cytometer (Becton‐Dickinson, San Diego, CA, USA), and cell cycle distribution was assessed with flowjo cell cycle platform v7.6.5 (Flowjo LCC, Ashland, OR, USA).

### 2.12 Bioinformatic analysis

Pathway enrichment analysis was performed using the Panther 2016 database in Enrichr domain (http://amp.pharm.mssm.edu/Enrichr). Pathways with *P* < 0.05 were considered as significant. Protein interactions were analyzed using STRING (https://string-db.org/).

### 2.13 Survival analysis

Data on gene expression and methylation of 470 individuals were downloaded from The Cancer Genome Atlas (TCGA) Skin Cutaneous Melanoma project. Of these, 79 individuals bearing primary tumors and 199 bearing secondary tumors (totalizing 278) had information on all covariates considered in this study and were used for the survival analyses. A multivariate Cox regression model was used to test the impact of gene expression and promoter methylation on patient overall survival. Age, tumor primary site, presence of metastasis in lymph nodes, ulceration, and Breslow depth value were used as covariates. Hazard ratios (HR) and corresponding 95% confidence intervals (CI) are shown. Statistical significance was set at *P* < 0.05. Kaplan–Meier survival curves were generated for genes with significant association with overall survival.

### 2.14 Statistical analysis

Data analysis was performed using graphpad prism 6 software (GraphPad Software, San Diego, CA, USA). Two and multiple group comparisons were analyzed by *t*‐test and ANOVA, respectively. For non‐normally distributed data, the Mann–Whitney test was used for two‐group comparison.

## 3 Results

### 3.1 Melanoma cell line 4C11+ exhibits highly aggressive and angiogenic phenotype *in vitro* and *in vivo*

**Figure 1:**
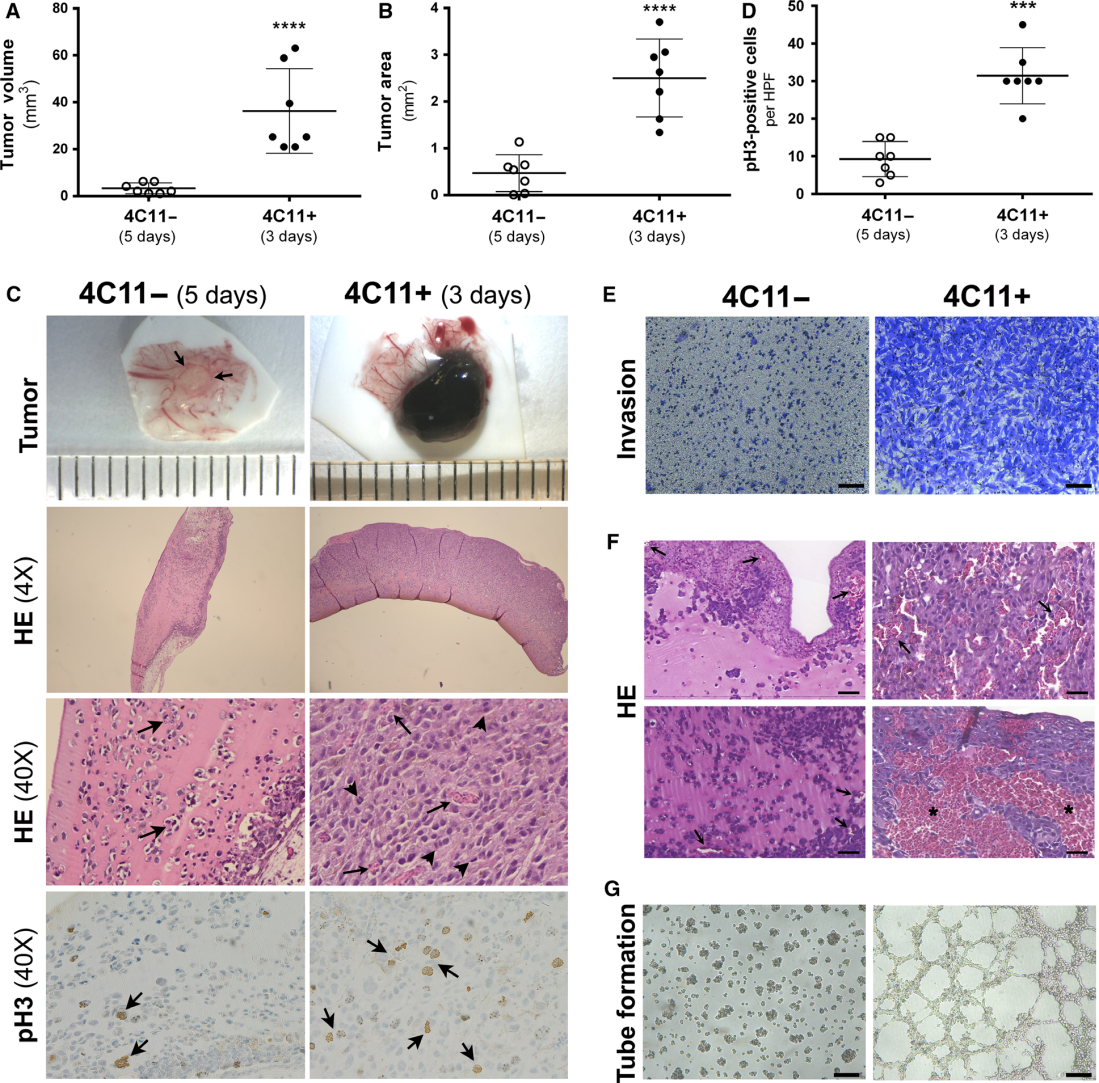
Melanoma cell line 4C11+ exhibits aggressive and proangiogenic phenotype *in vivo*. (A) Volume of tumors obtained in CAM assay. (B) Tumor area was quantified in Case Viewer software. Only tumor cells were quantified. Matrigel® and occasional blank spaces (cutting artifacts) were excluded from the total tumor area. (C) 4C11− and 4C11+ cells mixed with Matrigel® were applied on the CAM and incubated for 5 and 3 days, respectively. Representative macroscopic images, HE (4× and 40× magnification) and pH3 (40× magnification)‐stained sections of 4C11− and 4C11+ tumors are shown. In the 4C11− tumor *ex ovo* image, arrows highlight the small tumor. In HE images, arrows indicate blood vessels and arrowheads indicate mitotic cells. Each group consisted of at least seven specimens. (D) Quantification of pH3‐positive staining per HPF in CAM assay samples. (E) Representative images of Transwell invasion assay of 4C11− and 4C11+ cells after 48‐h incubation. Scale bar: 100 μm. (F) HE‐stained sections of 4C11− (3 days) and 4C11+ (5 days) tumors grown on the CAM were visualized by the case viewer software. Arrows and asterisks indicate blood vessels and hemorrhagic areas, respectively. Left panels show 4C11− tumors, which present well‐defined tumor vessels. Right panels show 4C11+ tumors, and the top right depicts extravasated blood cells adjacent to melanoma cells, while bottom right illustrates a large hemorrhagic area surrounding tumor cells. Scale bar: 50 μm. (G) Tube formation capability was assessed for 4C11− and 4C11+ cells seeded on Matrigel® after incubation of 16 h. Scale bar: 250 μm. Data are shown as mean ± SD, and statistical analysis was performed with Mann–Whitney *U*‐test (A) or *t*‐test (B‐C) (***P ≤ 0.001; **** *P* ≤ 0.0001).

4C11− and 4C11+ cells were engrafted onto the CAM and incubated for 3–5 days. 4C11‐ tumors grew at a slow rate, with only a small tumor mass being visible at day 3. However, 4C11+ cells at day 3 had already given rise to a tumor mass with an extensive network of blood vessels. At day 5, most of the embryos bearing 4C11+ tumors had died, probably due to the tumor aggressiveness. The 4C11+ tumors of chicken embryos that survived until day 5 were also removed and analyzed by HE staining; however, these samples harbored large hemorrhagic areas leaving hardly any tumor tissue (Fig. S1), which prevented further investigations. Thus, we decided to exploit 4C11− tumors on day 5 and 4C11+ tumors on day 3. In average, 4C11+ tumor volume was 11‐fold bigger than 4C11− tumors at the evaluated time points (Fig. 1A). For objective evaluation, HE‐stained tumor slices were scanned, which allowed the measurement of the actual tumor area by considering only tumor cell regions and excluding residual Matrigel®, CAM tissue, and large hemorrhagic areas. In average, the 4C11+ tumor area was 5‐fold bigger than the area of 4C11− tumors (Fig. 1B).

Hematoxylin and eosin histological analysis showed that in 4C11− tumors, neoplastic cells were separated by profuse eosinophilic material, such as Matrigel®. In these tumors, we found isolated intervening vessels and no evidence of CAM invasion (Fig. 1C, left panels). In contrast, 4C11+ tumors presented hyperchromatic nuclei, pleomorphic cells, extensive necrosis, and a hypervascularized and invasive tumor growth pattern (Fig. 1C, right panels). There was also a variable pigment formation in 4C11+ samples, which was clearly recognizable in the macroscopic tumor and in the HE staining. Abundant positive immunohistochemical staining for pH3, an important mitosis marker, was observed in 4C11+ cells (Fig. 1C, right panel). Staining quantification showed that 4C11+ tumors had in average 3 times more pH3‐positive cells [31/high‐power field (HPF); range 20–45] than 4C11− tumors (9/HPF; range 3–15; Fig. 1D), confirming the high proliferative index of 4C11+ cells (Fig. 1C). In addition, 4C11+ cells were shown to be highly invasive *in vitro* (Fig. 1E).

Scanned slices were also employed to better analyze tumor vascularization. All 4C11− tumor samples contained few and well‐defined intratumoral vessels; however, 4C11+ tumor samples presented large and irregular vessels and several hemorrhagic areas within the tumor masses (Fig. 1F). From seven analyzed tumors established from 4C11+ cells, four had large areas of hemorrhage and one was completely hemorrhagic (Fig. 1F,bottom right panel). In a tube formation assay *in vitro*, we could observe that 4C11+ cells formed an extensive network of capillary‐like structures, while 4C11− cells remained as single cells or formed irregularly shaped clusters of cells, and were, therefore, incapable to generate defined tubular vasculogenic mimicry (Fig. 1G).

### 3.2 Transcriptional analysis reveals alteration in angiogenesis‐related genes during melanoma progression

**Figure 2:**
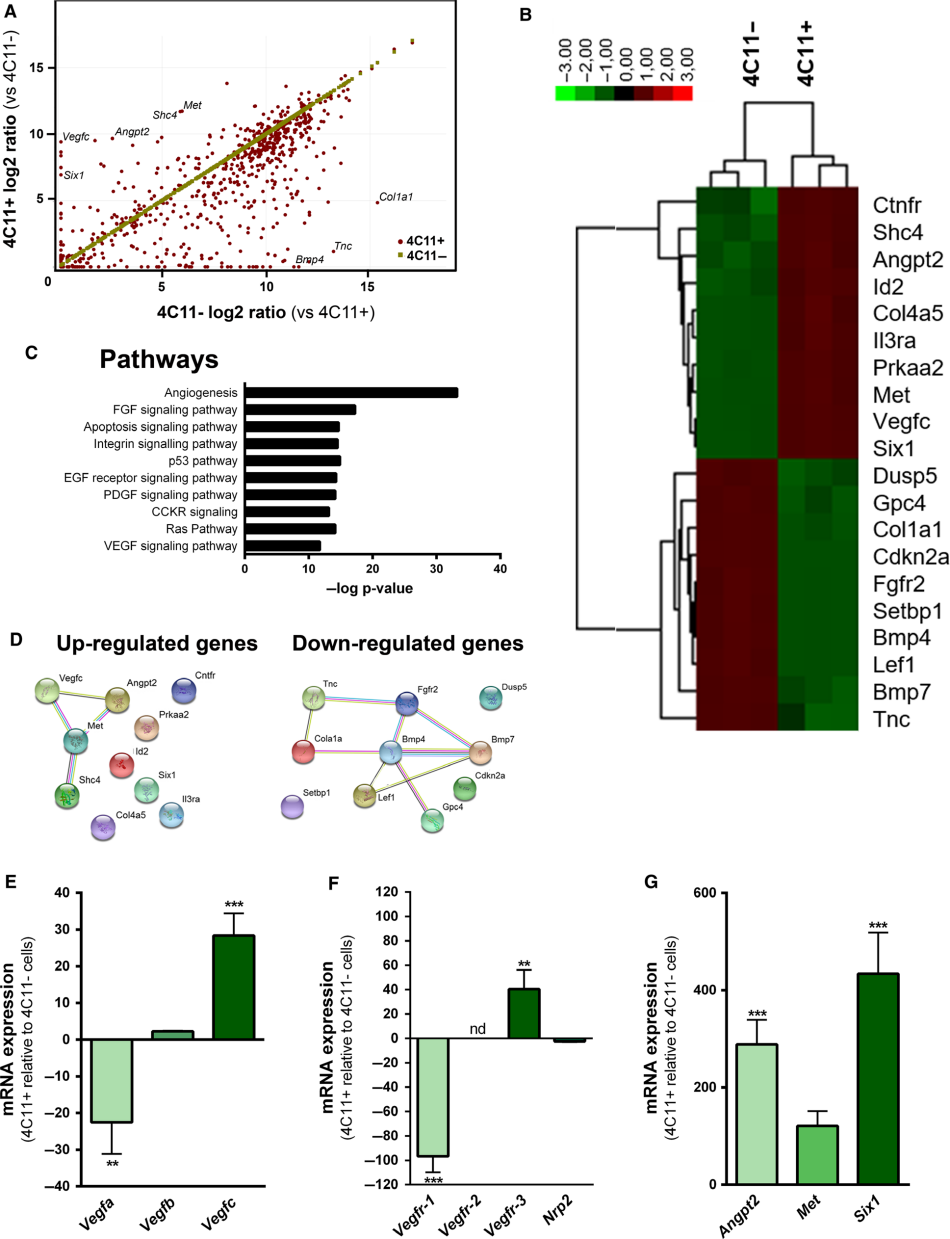
Transcriptional analysis reveals alteration in angiogenesis‐related genes during melanoma progression. (A) Scatter plot of 4C11− and 4C11+ mRNA expression assessed by the NanoString PanCancer Mouse Pathways Panel (*n* = 3). Each dot represents one gene. (B) Heatmap of unsupervised hierarchical clustering of 4C11− and 4C11+ cells with the 10 most upregulated and 10 most downregulated mRNA. Green and red represent, respectively, low and high mRNA expression level. (C) Ten most significantly enriched pathways of all dysregulated genes determined by the Panther database. (D) String analysis of the 10 most upregulated and downregulated genes in 4C11+ cells. (E–G) *Vegfa*,* Vegfb*,* Vegfc* (E); *Vegfr‐1*,* Vegfr‐2*,* Vegfr‐3*,* Npr2* (F); and *Angpt2*,* Met,* and *Six1* (G) mRNA expression was determined by RT‐qPCR in 4C11− and 4C11+ cells. Data are expressed as fold change normalized to *Actb* (*n* = 3). nd: not detected. Data represent mean ± SD, and statistical significance was evaluated by one‐way ANOVA followed by the Dunnett's *post hoc* test (**P ≤ 0.01; *** *P* ≤ 0.001).

To explore the molecular mechanisms responsible for the highly aggressive phenotype of 4C11+ cells, we profiled the expression pattern of 770 murine cancer‐related genes in 4C11− and 4C11+ cells with the NanoString nCounter technology. Using a twofold difference and *P* < 0.05 cutoff, we found 254 differentially expressed mRNA, of which 59 were significantly upregulated and 195 downregulated in 4C11+ in comparison with 4C11− cells (Fig. 2A and Tables S2 and S3). Unsupervised hierarchical clustering analysis demonstrated that 4C11− and 4C11+ cells could be distinguished accordingly to their mRNA expression profile (not shown). The 10 most up‐ and 10 most downregulated genes were hierarchically clustered and displayed in a heatmap (Fig. 2B). To gain further insight into the function of the dysregulated genes in 4C11+ cells, we performed a pathway enrichment analysis of all dysregulated genes using the Panther database. Among the most significant enriched pathways were FGF, PDGF, and VEGF signaling pathways, all directly associated with tumor progression and, most importantly, tumor metastasis and angiogenesis (Fig. 2C). KEGG pathway analysis also showed enrichment of tumor‐associated pathways (not shown). Interestingly, a String analysis showed that among the 10 most upregulated genes in 4C11+ compared with 4C11− cells, there is an association among the proteins encoded by *Vegfc* (the most upregulated gene), *Angpt2*,* Shc4*, and *Met*. The same analysis with the top 10 downregulated genes depicted an interaction network among *Tnc*,* Fgfr2*,* Cola1a*,* Bmp4*,* Bmp7*,* Lef1*, and *Gpc4* (Fig. 2D).

These data, in association with our functional assays, prompted us to study the angiogenesis process in these cells. Moreover, as *Vegfc* was the most dysregulated gene in the NanoString analysis, we decided to evaluate its family in more detail. We confirmed the differential expression of *Vegfa*,* Vegfb*,* Vegfc*, and their receptors, *Vegfr‐1*,* Vegfr‐2*,* Vegfr‐3,* and *Nrp2* by RT‐qPCR (Fig. 2E,F). We validated that *Vegfa* is downregulated in 4C11+ cells in comparison with 4C11− cells, while *Vegfb* and *Vegfc* are upregulated, the latter to a much higher extent. Considering the receptors, *Vegfr‐1* and *Nrp2* were downregulated in 4C11+ cells compared with 4C11− cells; *Vegfr‐2* could not be detected in either one of the cell lines and *Vegfr‐3* was highly upregulated in 4C11+ cells. Additionally, we confirmed the higher expression of *Angpt2*,* Met*, and *Six1* in 4C11+ cells (Fig. 2G).

### 3.3 Inhibition of VEGFC function decreases aggressiveness phenotype of metastatic 4C11+ cell line

**Figure 3:**
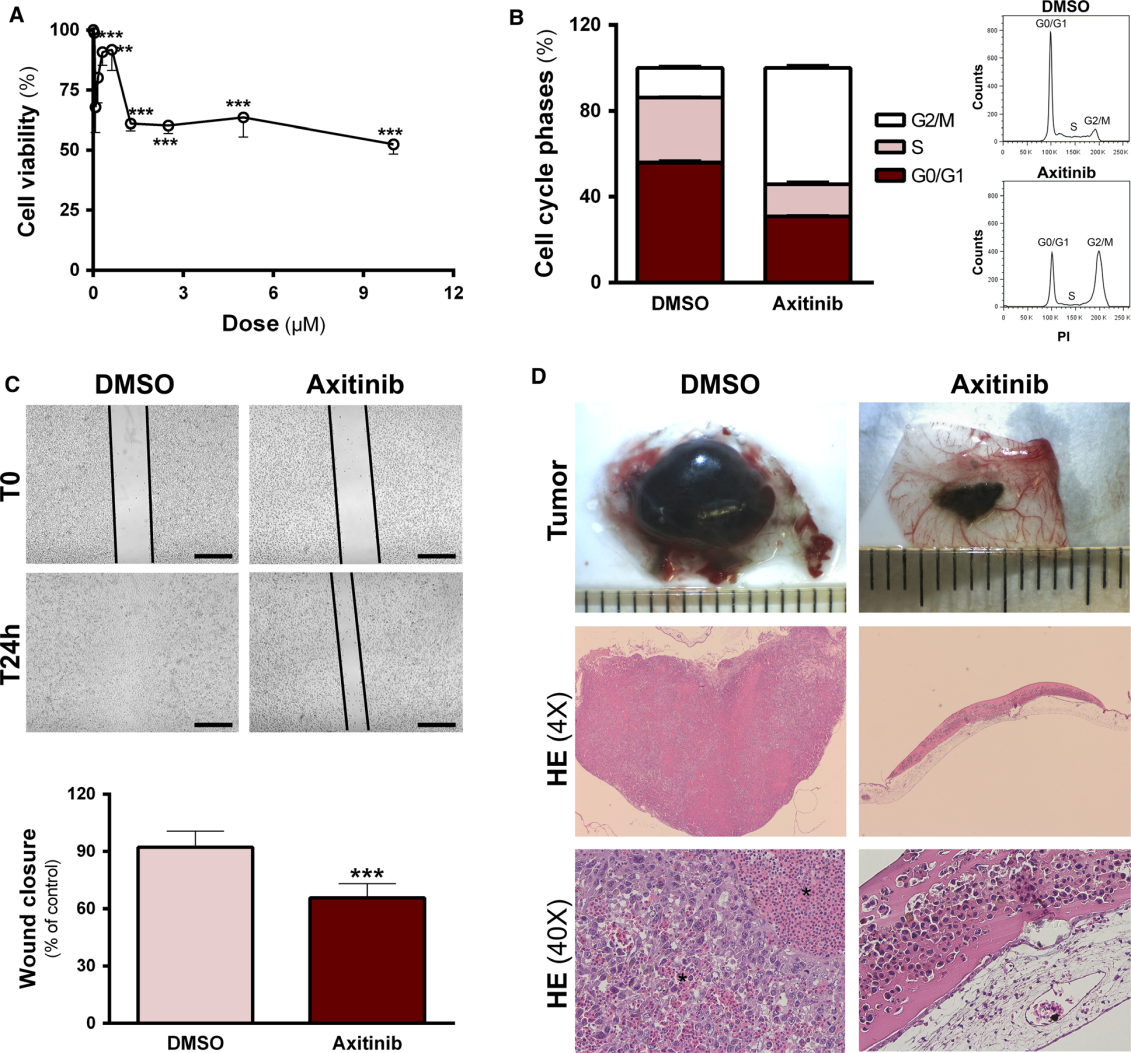
Inhibition of VEGFC function decreases aggressive phenotype of metastatic 4C11+ cell line. (A) Cell viability of 4C11+ cells treated with different concentrations of axitinib for 48 h was analyzed by MTT assay. Statistical significance was evaluated by one‐way ANOVA followed by the Dunnett's *post hoc* test. (B) Cell cycle distribution of 4C11+ cells after 48‐h treatment with 1 μm axitinib (*n* = 2) and representative cell cycle profile of 4C11+ cells treated with DMSO (control) or axitinib. (C) Migration of 4C11+ cells pretreated with 1 μm axitinib or the respective amount of DMSO was analyzed by wound healing assay for 24 h. Measurement of wound healing migration was performed in imagej Software and quantified by the equation: Migration % = [1 − (cell‐free area at *t*_24_/cell‐free area at *t*_0_) × 100] (*n* = 3). Statistical analysis was performed with *t*‐test. Scale bar: 50 μm. (D) Macroscopic *ex ovo* images and HE staining of tumors grown on the CAM. 4C11+ cells were pretreated *in vitro* with DMSO or 1 μm of axitinib for 48 h and then applied onto the CAM. Tumors were removed after 5 days of incubation on the CAM. Asterisks indicate hemorrhagic areas. Each group consisted of at least seven specimens. 4C11+ DMSO: 4C11+ cells treated with DMSO; 4C11+ axitinib: cells treated with axitinib. Data are displayed as mean ± SD (**P ≤ 0.01; *** *P* ≤ 0.001).

To better understand the impact of VEGFC in the aggressiveness of 4C11+ cells, we blocked the VEGFC pathway using axitinib, a potent and selective inhibitor of VEGF receptors. First, we evaluated the effects of axitinib on 4C11+ cells viability by a MTT dose–response analysis. Cells were treated with 40 nm–10 μm of axitinib for 48 h. Growth inhibition obtained using 1–5 μm of the drug was similar, ~ 40%, while 10 μm of the drug reduced cell viability to nearly 50%. For this, we decided to use the lowest effective dose of axitinib in 4C11+ cells, that is, 1 μm, for the subsequent experiments (Fig. 3A). Cell cycle analysis revealed that axitinib treatment induced a significant decrease of cells in the G1 phase, while massively increasing the G2/M population (13.4% in control cells to 52.2% in treated cells). There was no alteration in the sub‐G1 population (Fig. 3B). In wound healing assays, vehicle‐treated 4C11+ cells were able to close the wound after 24 h; however, axitinib‐treated cells had a 30% lower migration rate at that time point (Fig. 3C). After 48 h, there was no significant change in migration capability (data not shown). In the *in vivo* CAM assay, we observed that vehicle‐treated 4C11+ cells were able to develop massive tumors in 5 days, which were extremely invasive and presented extensive inflammatory infiltrates (Fig. 3D, left panels). On the other hand, axitinib‐treated 4C11+ cells generated only small tumor masses, in some cases failing to develop a tumor mass at all. HE staining depicts tumor cells surrounded by Matrigel® and clearly separated from the CAM, which was not infiltrated by the tumor cells (Fig. 3D, right panels). From 11 samples grown from axitinib‐pretreated 4C11+ cells, two were able to develop tumors, although much smaller than tumors generated by untreated 4C11+ cells. In one graft, cells were largely surrounded by Matrigel®, which was not observed in the control group, while in the other graft, the tumor growth was very similar to the DMSO‐treated group with cells invading the CAM and presenting high immune cell infiltration (Fig. S2).

### 3.4 High expression of *VEGFR‐3* and *ANGPT2* is associated with poor overall survival in melanoma patients

**Figure 4:**
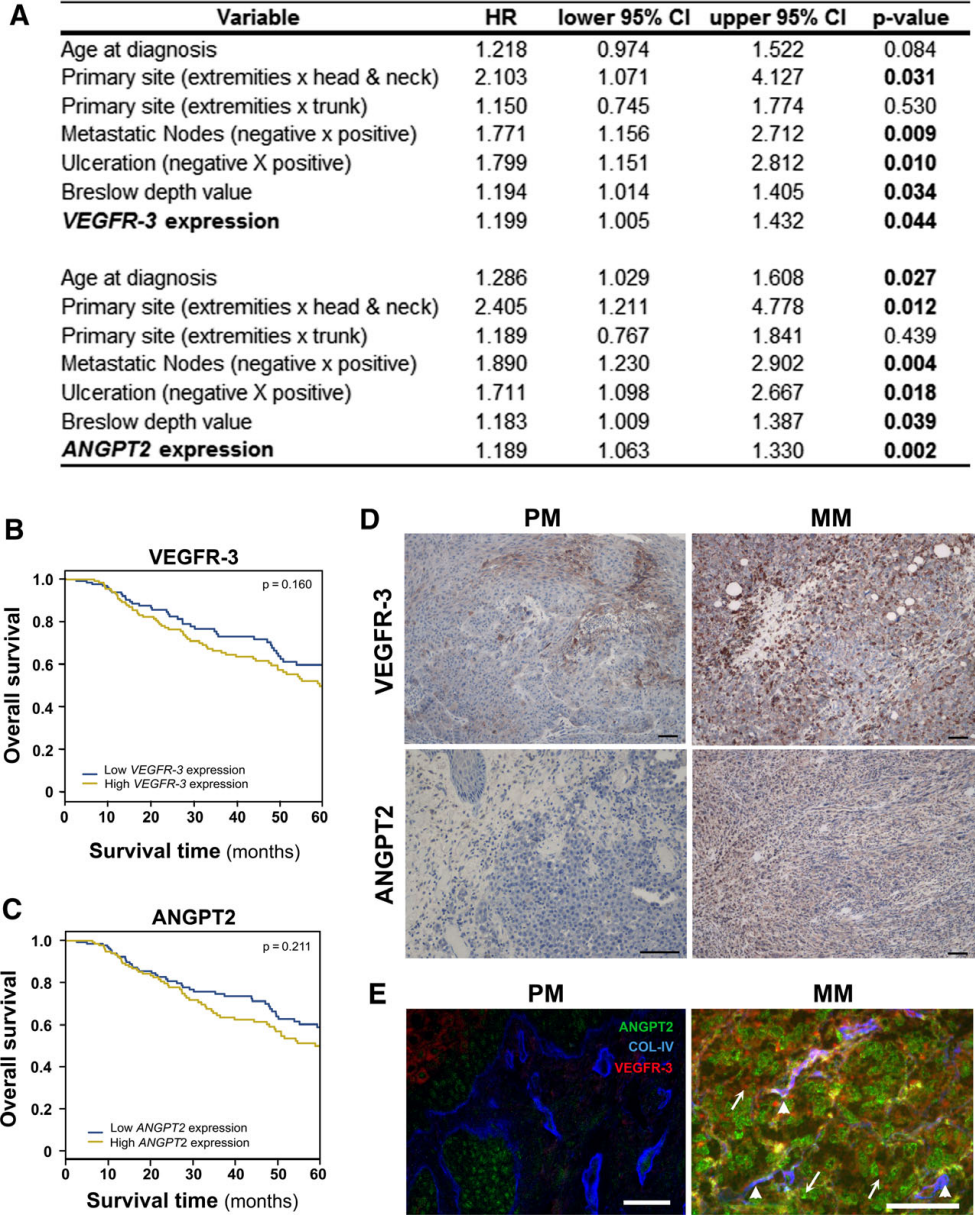
*VEGFR‐3* and *ANPGT2* expression correlates with overall survival in melanoma patients. (A) Multivariate analysis of clinicopathological features as well as *VEGFR‐3* and *ANPGT2* gene expression for predicting melanoma patient survival. HR and corresponding 95% CI are shown. (B–C) Kaplan–Meier survival analysis of melanoma patients according to *VEGFR‐3* (B) and *ANPGT2* (C) gene expression. Blue and yellow curves represent, respectively, low (bottom 50%) and high (top 50%) gene expression. Significance was determined by log‐rank test. (D) Representative IHC staining of VEGFR‐3 (upper panels) and ANGPT2 (lower panels) in primary (PM) and metastatic human melanoma tissue (MM) (*n* = 5). Scale bar: 100 μm. (E) Representative immunofluorescence of VEGFR‐3, ANGPT2, and Collagen IV in primary and metastatic human melanoma tissue (*n* = 3). Arrows indicate antigen expression in tumor cells and arrowheads, in blood vessels. Scale bar: 50 μm.

Cox multivariate analysis was performed to evaluate the association of gene expression and overall survival in melanoma patients. Expression of the following genes detected as upregulated in 4C11+ cells in comparison with 4C11− cells was analyzed individually: *VEGFC*,* VEGFR‐3*,* ANGPT2*,* MET,* and *SIX1*. After adjustment for age, tumor primary site, presence of metastasis in lymph nodes, ulceration, and Breslow depth value, high expression of *VEGFR‐3* (HR = 1.199; *P*‐value = 0.044) and *ANGPT2* (HR = 1.189; *P*‐value = 0.002) was shown to be predictors of shorter overall survival (Fig. 4A). Kaplan–Meier curves of *VEGFR‐3* and *ANGPT2* are shown (Fig. 4B,C). The expression of VEGFR‐3 and ANGPT2 was also evaluated by IHC and MELC staining in primary and metastatic human melanoma tissue. In the IHC, only the staining of melanocytes and melanoma cells were evaluated. Skin and colon tissue were used as negative controls (Fig. S3). The average positive staining intensity of the antigens was weak in primary samples (IRS: VEGFR‐3 = 3.6; ANGPT2 = 3.1) and moderate in the metastatic specimens (IRS: VEGFR‐3 = 5.3; ANGPT2 = 5.6; Fig. 4D). In the MELC technique, we also stained blood vessels with Collagen type IV. This assay confirmed the high expression of ANGPT2 and VEGFR‐3 in metastatic melanomas and showed these antigens are expressed by both the tumor and endothelial cells. While VEGFR‐3 was mainly detected in the tumor vasculature, ANGPT2 was highly expressed by the tumor cells (Fig. 4E).

### 3.5 Expression of *Vegfc*,* Angpt2,* and *Six1* is epigenetically regulated in murine melanoma cell lines

**Figure 5:**
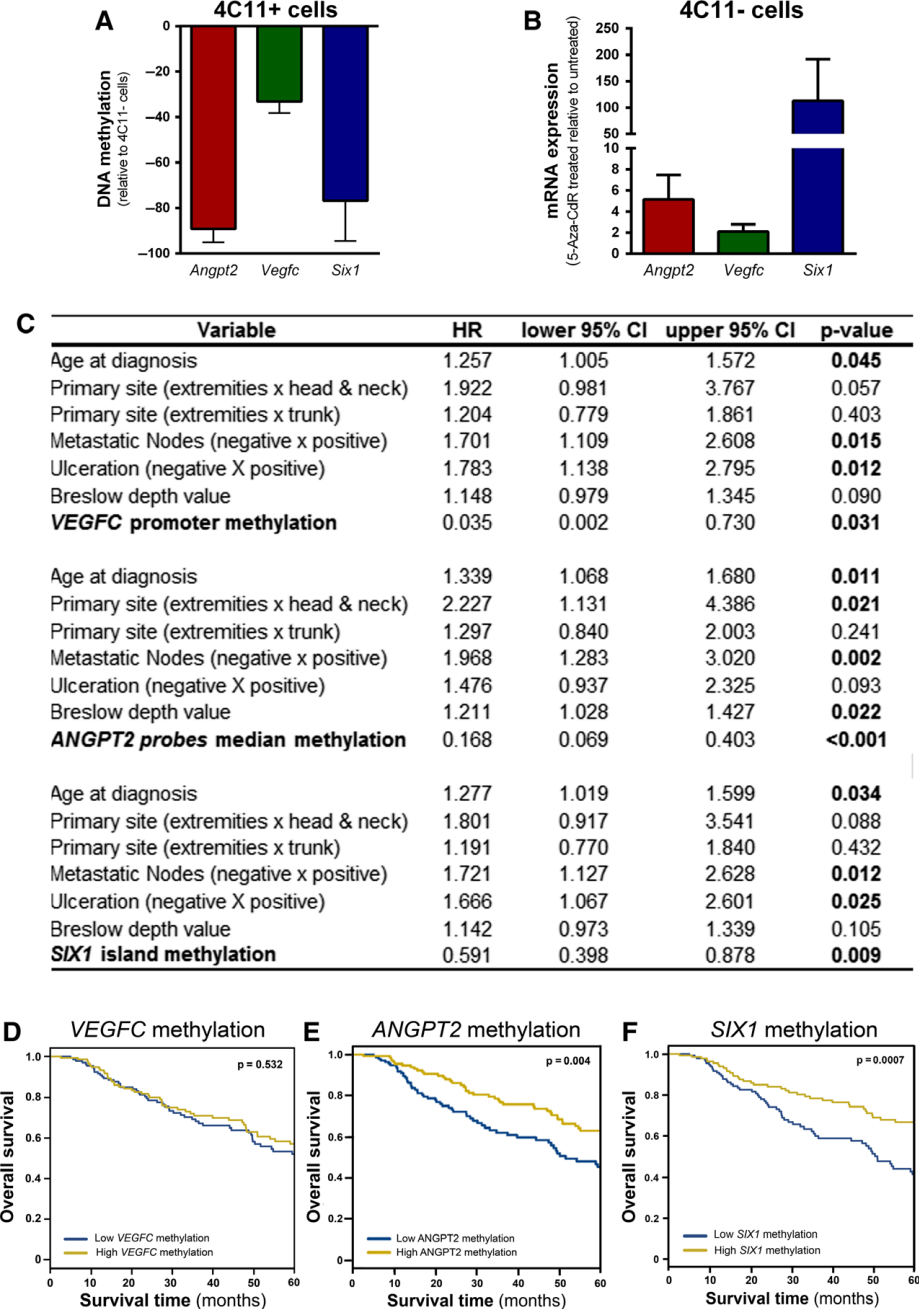
*Vegfc*,* Angpt2,* and *Six1* promoter methylation is associated with overall survival in melanoma patients. (A) Average methylation of CpGs differentially methylated of *Vegfc*,* Angpt2,* and *Six1* in 4C11+ cells compared with 4C11− cells as evaluated by methylome sequencing (*P*‐value < 0.01). (B) mRNA expression of *Vegfc, Angpt2,* and *Six1* in 4C11− cells treated with 5‐Aza‐CdR in comparison with 4C11− cells treated with vehicle (DMSO) as assessed by RT‐qPCR. Data are expressed as fold change normalized to *Actb* (*n* = 3). Data are displayed as mean ± SD. (C) Multivariate analysis was performed to correlate clinicopathological features and *VEGFC*,* ANPGT2,* or *SIX1* promoter methylation to melanoma patient survival. HR and corresponding 95% CI are shown. (D–E) Kaplan–Meier survival analysis of melanoma patients according to *VEGFC* (D), *ANPGT2* (E), and *SIX1* (F) promoter methylation. Blue and yellow curves represent, respectively, low (bottom 50%) and high (top 50%) DNA methylation. Significance was determined by the log‐rank test.

The DNA methylation status of *Vegfc*,* Vegfr‐3*,* Angpt2*,* Met,* and *Six1* was verified in a methylome sequencing data from enhanced reduced representation bisulfite sequencing (Rius *et al*., in preparation). CpGs distant up to 1500 nucleotides upstream and 250 nucleotides downstream from the transcription start site (TSS) were analyzed, and a CpG site was considered to be differentially methylated if it presented a minimum of 25% difference in methylation with a *P*‐value ≤ 0.01. The number of CpGs differentially methylated in *Vegfc*,* Angpt2,* and *Six1* promoters was four (distant from 147–222 nucleotides downstream of the TSS), 11 (located from 1129 to 1370 nucleotides upstream of the TSS), and 72 (−1499 to +203 distant from the TSS), respectively. These CpGs sites had in average 33%, 87%, and 76% lower methylation in 4C11+ cells than in 4C11− cells, respectively (Fig. 5A and Table S4). In the analyzed region, *Vegfr‐3* and *Met* did not show any differential methylation in 4C11+ in comparison with 4C11− cells. We then treated 4C11− cells, which express low levels of *Vegfc, Angpt2,* and *Six1*, with the DNA methyltransferase inhibitor 5‐Aza‐CdR and verified their expression by RT‐qPCR. Demethylation caused by the treatment enhanced the expression of the three genes analyzed, most prominently of *Angpt2* and *Six1* (Fig. 5B).

### 3.6 Promoter methylation of *VEGFC*,* ANGPT2,* and *SIX1* is associated with poor prognosis in melanoma patients

As we observed an epigenetic regulation of *Vegfc, Angpt2,* and *Six1* in our mouse melanoma model, we analyzed whether the promoter methylation of these genes could predict overall survival in melanoma patients by a Cox multivariate analysis. Promoter CpG island and shore methylation of *VEGFC* (HR_island + shore_ = 0.035; *P*‐value = 0.031) and *SIX1* (HR_island_ = 0.591; *P*‐value = 0.009) were analyzed and found to be significantly associated with survival. *ANGPT2* promoter does not contain a CpG island; therefore, we analyzed single CpGs shown to be hypomethylated in 4C11+ cells and that had also been previously evaluated in chronic lymphocytic leukemia (Martinelli *et al*., 2013). The average DNA methylation of these CpGs was associated with overall survival (HR = 0.1677; *P*‐value < 0.001). In all cases, decreased methylation was associated with shorter survival of melanoma patients (Fig. 5C). Kaplan–Meier curves are shown (Fig. 5D–F).

## 4 Discussion

Metastasis is closely associated with high mortality rate in melanoma patients. Therefore, melanoma cell dissemination and the related molecular mechanisms still need to be elucidated in more detail. New diagnostic and prognostic markers are also important to reach a better clinical outcome and mortality reduction. To study melanoma progression, we used a linear murine progression model in which metastatic 4C11+ cells arose from nonmetastatic 4C11− cells following P53 expression loss (Souza *et al*., 2012).

To better analyze the cancer properties of 4C11− and 4C11+ cells, we performed the CAM assay, which allows the study of several hallmarks of cancer, as proliferation, invasion, metastasis, and angiogenesis (Lokman *et al*., 2012; Muenzner *et al*., 2018). Here, we could observe a clear distinction between the two cell lines. *In vivo*, 4C11+ cells gave rise to larger tumors, which were highly proliferative and vascularized. On the other hand, 4C11− tumors displayed small well‐defined tumor masses with localized tumor vessel infiltration. We also observed 4C11+ cells were highly invasive *in vitro*. These data are consistent and enrich our previous results showing different growth rate and metastasis capability of 4C11− and 4C11+ cells in a mouse model (Souza *et al*., 2012). Interestingly, 4C11+ cells also developed an extensive network of capillary‐like structures in an *in vitro* tube formation assay, indicating a high vascular mimicry (VM) capacity. These 3D tube‐like structures consist of tumor cells and extracellular matrix and are endothelial cell‐free. VM can function as an alternative supplier of blood to tumor masses, independently of angiogenesis, and can contribute to metastasis (Chung and Mahalingam, 2014). The high VM capability of 4C11+ cells is consistent with their excessive bleeding observed in the CAM assay and can contribute to it.

Investigation of the expression pattern of cancer‐related genes in 4C11− and 4C11+ cells revealed that *Vegfc* and *Angpt2* were among the top 10 upregulated genes. Both are important regulators of the vascular phenotype in tumors (Kim *et al*., 2009). A pathway‐based analysis of all dysregulated genes revealed enrichment of several angiogenic pathways, including FGF, PDGF, and VEGF signaling. In String analysis, we found that VEGFC, ANGPT2, MET, and SHC4 proteins interact, which is a further support of their possible role in the 4C11+ cells aggressive phenotype. Several downregulated genes were shown to have a strong protein interaction, but most of these genes, such as *Fgfr2*,* Tnc,* and *Lef1*, have been positively associated with tumor progression (Katoh and Nakagama, 2013; Murakami *et al*., 2001; Shao *et al*., 2015), suggesting their downregulation is not involved in 4C11+ cells aggressive phenotype. Conversely, *Bmp4* is a known inhibitor of angiogenesis (Tsuchida *et al*., 2014); thus, its downregulation probably contributes to 4C11+ tumor vascularization. Compared with 4C11− cells, 4C11+ cells presented upregulation of *Vegfb*,* Vegfc*, and *Vegfr‐3*. Importantly, the expression of VEGF receptors by tumor cells is a known indicator of high aggressiveness (Mouawad *et al*., 2009). As VEGFC has a high affinity for VEGFR‐3, our data indicate that the VEGF pathway effector in 4C11+ cells must be VEGFC by binding and activating VEGFR‐3 in an autocrine signaling, which is classically recognized to be involved in lymphangiogenesis (Mouawad *et al*., 2009).

As oxygen and nutrient supply are mandatory for tumor growth, the VEGF pathway, angiogenesis, and lymphangiogenesis are not only essential for the metastasis process, but also for the progression of solid tumors beyond a critical size (Alitalo *et al*., 2013). Indeed, recently VEGFC and VEGFD have also been reported to regulate the inflammatory tumor microenvironment, which regulates early stages of tumor growth (Alitalo *et al*., 2013). Nonetheless, high levels of VEGFC correlate with melanoma metastasis to lymph nodes, which is one of the most important markers of poor prognosis for melanoma patients (Tímár *et al*., 2016). It has also been demonstrated that VEGFC and lymphangiogenesis can contribute to the development of distant metastasis (Ma *et al*., 2018).

We also confirmed the expression of *Angpt2*,* Met,* and *Six1*; genes involved in metastasis and angiogenesis. ANGPT2 is a secreted growth factor that sensitizes endothelial cells to different proangiogenic factors, such as VEGFs, and it has been shown to promote tumor metastasis, angiogenesis, and lymphangiogenesis (Holopainen *et al*., 2012). SIX1 was recently shown to have pro‐oncogenic and metastatic properties in different tumors (Coletta *et al*., 2008; Wang *et al*., 2012). Interestingly, to the best of our knowledge, there are no studies available in melanoma. In addition, SIX1 is capable of inducing lymphangiogenesis by increasing VEGFC expression (Liu *et al*., 2014; Wang *et al*., 2012). The high expression of *Angpt2, Six1,* and *Vegfc* in 4C11+ cells suggests that they might contribute together to the vascular phenotype of these cells.

Then, we blocked the VEGF pathway in 4C11+ cells using axitinib, a potent and selective inhibitor of VEGFRs (Zhang *et al*., 2014). This drug competitively binds to the intracellular ATP site domain of the receptors, stabilizing them in an inactive conformation and therefore inhibiting downstream signal transduction (Gross‐Goupil *et al*., 2013). The treatment induced a significant G2/M arrest, reduced 4C11+ cells ability to migrate *in vitro* and to develop tumors *in vivo*. As axitinib is a classical inhibitor of angiogenesis, most studies analyzed the effects of the drug in an already developed tumor (He *et al*., 2014; Zhang *et al*., 2014), but we aimed to examine the ability of cells previously treated with the drug to develop a tumor. While 4C11+ control cells developed big tumor masses, axitinib‐pretreated 4C11+ cells only gave rise to small, noninvasive tumors. This is presumably due to the inhibition of VEGFC signaling—the only VEGF ligand expressed in these cells—which leads to an impaired tumor development. Indeed, axitinib has been reported to have antitumor activity in highly angiogenic tumors (Fruehauf *et al*., 2011).

Axitinib has previously been shown to induce senescence in gastric cancer (He *et al*., 2014) and glioma cells (Morelli *et al*., 2016), a phenotype suggested by the G2/M arrest observed in 4C11+ ‐treated cells. Although senescence can influence tumor growth (Rodier and Campisi, 2011), former studies have shown that major axitinib effects are due to the VEGF pathway blockade. Nonetheless, the profound abrogation of tumor growth caused by the VEGF receptors blockage, and consequent inhibition of VEGFC function, suggests the VEGFC pathway has an important role in the aggressive phenotype of 4C11+ cells. Further studies of our group should elucidate whether lymphangiogenesis is involved in 4C11+ tumor cells dissemination.

To evaluate the translational relevance of our findings, we performed a Cox multivariate analysis for the expression of *VEGFC*,* ANGPT2*,* MET, SIX1*, and *VEGFR‐3*. After adjustment for the covariates, high expression of *VEGFR‐3* and *ANGPT2* were both independent predictors of poor prognosis. Kaplan–Meier analysis illustrated that patients with low and high expression of these genes have distinct survival curves, although log‐rank tests were not significant. Reinforcing these data, IHC and MELC staining demonstrated that tissues obtained from metastatic melanoma patients, which are known to have a worse prognosis, have higher expression levels of VEGFR‐3 and ANGPT2 compared with tissues obtained from patients with primary melanoma. In the samples analyzed by the MELC technique, VEGFR‐3 was mainly detected in the tumor vasculature, while ANGPT2 was highly expressed by the tumor cells. Although these molecules are predominantly expressed by endothelial cells, recent studies have reported their expression by tumor cells (Streit and Detmar, 2003; Su *et al*., 2008a,2008b). Notably, VEGFR‐3 and ANGPT2 were previously observed to be expressed in melanoma tumor cells (Helfrich *et al*., 2009; Mouawad *et al*., 2009).

vascular endothelial growth factor receptor‐3 expression was already shown to be a prognostic marker of disease‐free survival in gastric adenocarcinoma (Jüttner *et al*., 2006), but there are only a few studies in melanoma. Soluble VEGFR‐3 has been reported to be associated with disease‐free survival but not overall survival in melanoma patients (Mouawad *et al*., 2009). Moreover, *ANGPT2* expression was correlated with overall patient survival in colorectal and breast cancer (Hong *et al*., 2017; Sfiligoi *et al*., 2003). In melanoma, one study demonstrated high levels of circulating ANGPT2 to be associated with poor patient overall survival (Helfrich *et al*., 2009). To our knowledge, we are the first to show that *ANGPT2* (HR = 1.189; *P* = 0.002) and *VEGFR‐3* (HR = 1.999; *P* = 0.044) are independent predictors of prognosis in melanoma patients.

Furthermore, we detected that *Vegfc*,* Angpt2,* and *Six1* promoters were hypomethylated in 4C11+ cells compared with 4C11− cells. Importantly, the methylation status of these genes was inversely correlated to their mRNA expression levels. Indeed, treatment of 4C11− cells with 5‐Aza‐CdR increased the expression of the three genes, suggesting that DNA methylation regulates their transcription. Interestingly, we found low levels of *VEGFC*,* ANGPT2,* and *SIX1* promoter methylation are independent prognostic factors of poor patient survival. *VEGFC* expression has previously been shown to predict melanoma patient survival (Boone *et al*., 2008; Liu *et al*., 2008), and its expression has been shown to be regulated by DNA methylation in gastric cancer (Matsumura *et al*., 2007). Consistently, VEGFC promoter methylation was reported to be associated with progression‐free survival in ovarian cancer (Dai *et al*., 2013). Concerning *ANGPT2*, the methylation status of 6 CpGs near the gene transcription site (four of which were analyzed in this study) has already been shown to predict overall survival of chronic lymphocytic leukemia patients (Martinelli *et al*., 2013). Several studies have reported that SIX1 overexpression is frequently associated with poor patient prognosis in various malignancies, as colorectal cancer (Kahlert *et al*., 2015) and glioma (Zhang and Xu, 2017), however not in melanoma. Besides, the mechanisms responsible for the high expression of SIX1 have been poorly investigated. Methylation of *SIX1* promoter has previously been reported as a transcription regulatory mechanism in the porcine and bovine muscle (Wei *et al*., 2018; Wu *et al*., 2013). To our knowledge, this is the first report to show *VEGFC* (HR = 0.035; *P* = 0.031), *ANGPT2* (HR = 0.168; *P* < 0.001), and *SIX1* (HR = 0.591; *P* = 0.009) promoter methylation as prognostic markers for melanoma patient survival.

## 5 Conclusion

In summary, we found that the VEGFC pathway is highly correlated with tumor aggressiveness in our murine model. Moreover, we identified *VEGFR‐3* and *ANGPT2* expression, as well as *VEGFC, ANGPT2,* and *SIX1* promoter methylation, as independent prognostic factors for overall survival in melanoma patients, showing the high translational relevance of our findings obtained in a murine model.

## Conflict of interest

The authors declare no conflict of interest.

## Author contributions

ACM, JKM, RSS, and MGJ conceived and designed the study, interpreted the data, and participated in manuscript preparation. ACM performed most experiments and drafted the manuscript. ACM and JKM performed the CAM assays. FER carried out the methylome analysis. FA and AF performed the survival analysis. CO performed the IHC and MELC analysis on human specimens. CIG digitalized the CAM tissue sections for CaseViewer evaluation. AH and AG performed the CAM histological examinations. All authors read and approved the final manuscript.

## Authors’ information

The present work was performed in partial fulfillment of the requirements for obtaining the PhD degree ‘Dr.rer. nat.’ at the FAU Erlangen‐Nürnberg for ACM. ACM was a Cotutelle student at FAU and UNIFESP being integrated in the IZKF doctoral program of the FAU.

## Supporting information


**Fig. S1.** Representative macroscopic images and HE stained tissue sections (4× magnification) of 4C11+ tumors grown on the CAM for 5 days.
**Fig. S2.** Representative macroscopic image and HE staining (4× and 40× magnification) of tumors grown on the CAM. 4C11+ cells were pretreated *in vitro* with 1 μm of Axitinib for 48 h and applied onto the CAM. Tumors grown were removed after 5 days.
**Fig. S3.** Representative images of VEGFR‐3 and ANGPT2 staining in normal skin and colon, which were used as negative controls of the IHC staining.
**Table S1.** Primers sequences for RT‐qPCR reactions.
**Table S2.** mRNA upregulated in 4C11+ cells in comparison to 4C11− cells as assessed by the NanoString Panel.
**Table S3.** mRNA downregulated in 4C11+ cells in comparison to 4C11− cells as assessed by the NanoString Panel.
**Table S4.** CpGs differentially methylated of *Vegfc*,* Angpt2* and *Six1* promoters regions in 4C11+ cells compared to 4C11− cells evaluated by ERRBS.

## Data Availability

The TCGA Skin Cutaneous Melanoma project data can be found in https://portal.gdc.cancer.gov/projects/TCGA-SKCM.

## References

[mol212501-bib-0001] Adler NR , Haydon A , McLean CA , Kelly JW and Mar VJ (2017) Metastatic pathways in patients with cutaneous melanoma. Pigment Cell Melanoma Res 30, 13–27. 27900851 10.1111/pcmr.12544

[mol212501-bib-0002] Alitalo AK , Proulx ST , Karaman S , Aebischer D , Martino S , Jost M , Schneider N , Bry M and Detmar M (2013) VEGF‐C and VEGF‐D blockade inhibits inflammatory skin carcinogenesis. Can Res 73, 4212–4221. 10.1158/0008-5472.CAN-12-4539 23695550

[mol212501-bib-0003] Bolander A , Wagenius G , Larsson A , Brattström D , Ullenhag G , Hesselius P , Ekman S and Bergqvist M (2007) The role of circulating angiogenic factors in patients operated on for localized malignant melanoma. Anticancer Res 27, 3211–3217. 17970063

[mol212501-bib-0004] Boone B , Blokx W , De Bacquer D , Lambert J , Ruiter D and Brochez L (2008) The role of VEGF‐C staining in predicting regional metastasis in melanoma. Virchows Arch 453, 257–265. 18679715 10.1007/s00428-008-0641-6

[mol212501-bib-0005] Cao W , Fan R , Yang W and Wu Y (2014) VEGF‐C expression is associated with the poor survival in gastric cancer tissue. Tumor Biol 35, 3377–3383. 10.1007/s13277-013-1445-0 24307624

[mol212501-bib-0006] Chung HJ and Mahalingam M (2014) Angiogenesis, vasculogenic mimicry and vascular invasion in cutaneous malignant melanoma‐implications for therapeutic strategies and targeted therapies. Expert Rev Anticancer Ther 14, 621–639. 24506089 10.1586/14737140.2014.883281

[mol212501-bib-0007] Clark JI , Singh J , Ernstoff MS , Lao CD , Flaherty LE , Logan TF , Curti B , Agarwala SS , Taback B , Cranmer L *et al* (2018) A multi‐center phase II study of high dose interleukin‐2 sequenced with vemurafenib in patients with BRAF‐V600 mutation positive metastatic melanoma. J ImmunoTher Cancer 1–8. 30053905 10.1186/s40425-018-0387-x PMC6062934

[mol212501-bib-0008] Coletta RD , Christensen KL , Micalizzi DS , Jedlicka P , Varella‐Garcia M and Ford HL (2008) Six1 overexpression in mammary cells induces genomic instability and is sufficient for malignant transformation. Cancer Res 68, 2204–2214. 18381426 10.1158/0008-5472.CAN-07-3141

[mol212501-bib-0009] Dai W , Zeller C , Masrour N , Siddiqui N , Paul J and Brown R (2013) Promoter CpG island methylation of genes in key cancer pathways associates with clinical outcome in high‐grade serous ovarian cancer. Clin Cancer Res 19, 5788–5797. 23965899 10.1158/1078-0432.CCR-13-1217 PMC4913863

[mol212501-bib-0010] Dumitru CA , Bankfalvi A , Gu X , Eberhardt WE , Zeidler R , Lang S and Brandau S (2013) Neutrophils activate tumoral CORTACTIN to enhance progression of orohypopharynx carcinoma. Front Immunol 4, 1–11. 23423155 10.3389/fimmu.2013.00033 PMC3574976

[mol212501-bib-0011] Fruehauf J , Lutzky J , McDermott D , Brown CK , Meric JB , Rosbrook B , Shalinsky DR , Liau KF , Niethammer AG , Kim S *et al* (2011) Multicenter, Phase II study of axitinib, a selective second‐generation inhibitor of vascular endothelial growth factor receptors 1, 2, and 3, in patients with metastatic melanoma. Clin Cancer Res 17, 7462–7469. 21976544 10.1158/1078-0432.CCR-11-0534

[mol212501-bib-0012] Gross‐Goupil M , François L , Quivy A and Ravaud A (2013) Axitinib: a review of its safety and efficacy in the treatment of adults with advanced renal cell carcinoma. Clin Med Insights Oncol 7, 269–277. 24250243 10.4137/CMO.S10594 PMC3825605

[mol212501-bib-0013] He Q , Gao J , Ge S , Wang T , Li Y , Peng Z , Li Y and Shen L (2014) Axitinib alone or in combination with chemotherapeutic drugs exerts potent antitumor activity against human gastric cancer cells *in vitro* and *in vivo* . J Cancer Res Clin Oncol 140, 1575–1583. 24804814 10.1007/s00432-014-1693-4 PMC11823817

[mol212501-bib-0014] Helfrich I , Edler L , Sucker A , Thomas M , Christian S , Schadendorf D and Augustin HG (2009) Angiopoietin‐2 levels are associated with disease progression in metastatic malignant melanoma. Clin Cancer Res 15, 1384–1392. 19228739 10.1158/1078-0432.CCR-08-1615

[mol212501-bib-0015] Holopainen T , Saharinen P , D'Amico G , Lampinen A , Eklund L , Sormunen R , Anisimov A , Zarkada G , Lohela M , Heloterä H *et al* (2012) Effects of angiopoietin‐2‐blocking antibody on endothelial cell‐cell junctions and lung metastasis. J Natl Cancer Inst 104, 461–475. 22343031 10.1093/jnci/djs009 PMC3309130

[mol212501-bib-0016] Hong S , Jung HI , Ahn TS , Kim HJ , Lee KT , Baek MJ and Bae SB (2017) Expressions and clinical significances of angiopoietin‐1, angiopoietin‐2, and tie‐2 receptor in patients with colorectal cancer. Ann Coloproctol 33, 9–15. 28289658 10.3393/ac.2017.33.1.9 PMC5346784

[mol212501-bib-0017] Jüttner S , Wissmann C , Jöns T , Vieth M , Hertel J , Gretschel S , Schlag PM , Kemmner W and Höcker M (2006) Vascular endothelial growth factor‐D and its receptor VEGFR‐3: two novel independent prognostic markers in gastric adenocarcinoma. J Clin Oncol 24, 228–240. 16344322 10.1200/JCO.2004.00.3467

[mol212501-bib-0018] Kahlert C , Lerbs T , Pecqueux M , Herpel E , Hoffmeister M , Jansen L , Brenner H , Chang‐Claude J , Bläker H , Kloor M *et al* (2015) Overexpression of SIX1 is an independent prognostic marker in stage I – III colorectal cancer. Int J Cancer 137, 2104–2113. 25951369 10.1002/ijc.29596

[mol212501-bib-0019] Katoh M and Nakagama H (2013) FGF receptors: cancer biology and therapeutics. Med Res Rev 1–21. 10.1002/med.21288 23696246

[mol212501-bib-0020] Kim HZ , Jung K , Kim HM , Cheng Y and Koh GY (2009) A designed angiopoietin‐2 variant, pentameric COMP‐Ang2, strongly activates Tie2 receptor and stimulates angiogenesis. Biochim Biophys Acta 1793, 772–780. 19339208 10.1016/j.bbamcr.2009.01.018

[mol212501-bib-0021] Li P , He Q , Luo C and Qian L (2015) Diagnostic and prognostic potential of serum angiopoietin‐2 expression in human breast cancer. Int J Clin Exp Pathol 8, 660–664. 25755760 PMC4348815

[mol212501-bib-0022] Liu D , Li L , Zhang XX , Wan DY , Xi BX , Hu Z , Ding WC , Zhu D , Wang XL , Wang W *et al* (2014) SIX1 promotes tumor lymphangiogenesis by coordinating TGFβ signals that increase expression of VEGF‐C. Cancer Res 74, 5597–5607. 25142796 10.1158/0008-5472.CAN-13-3598

[mol212501-bib-0023] Liu B , Ma J , Wang X , Su F , Li X , Yang S , Ma W and Zhang Y (2008) Lymphangiogenesis and its relationship with lymphatic metastasis and prognosis in malignant melanoma. Anat Rec 291, 1227–1235. 10.1002/ar.20736 18561194

[mol212501-bib-0024] Lokman NA , Elder AS , Ricciardelli C and Oehler MK (2012) Chick chorioallantoic membrane (CAM) assay as an *in vivo* model to study the effect of newly identified molecules on ovarian cancer invasion and metastasis. Int J Mol Sci 13, 9959–9970. 22949841 10.3390/ijms13089959 PMC3431839

[mol212501-bib-0025] Ma Q , Dieterich LC , Ikenberg K , Bachmann SB , Mangana J , Proulx ST , Amann VC , Levesque MP , Dummer R , Baluk P *et al* (2018) Unexpected contribution of lymphatic vessels to promotion of distant metastatic tumor spread. Sci Adv 1, 1–10. 10.1126/sciadv.aat4758 PMC6082649 30101193

[mol212501-bib-0026] Martinelli S , Kanduri M , Maffei R , Fiorcari S , Bulgarelli J , Marasca R and Rosenquist R (2013) ANGPT2 promoter methylation is strongly associated with gene expression and prognosis in chronic lymphocytic leukemia. Epigenetics 8, 720–729. 23803577 10.4161/epi.24947 PMC3781191

[mol212501-bib-0027] Matsumoto T and Claesson‐Welsh L (2001) VEGF receptor signal transduction. Sci STKE 112, RE21. 10.1126/stke.2001.112.re21 11741095

[mol212501-bib-0028] Matsumura S , Oue N , Mitani Y , Kitadai Y and Yasui W (2007) DNA demethylation of Vascular endothelial growth factor‐C is associated with gene expression and its possible involvement of lymphangiogenesis in gastric cancer. Int J Cancer 120, 1689–1695. 17230534 10.1002/ijc.22433

[mol212501-bib-0029] Morelli M , Amantini C , Nabissi M , Cardinali C , Santoni M , Bernardini G , Santoni A and Santoni G (2016) Axitinib induces senescence‐associated cell death and necrosis in glioma cell lines: the proteasome inhibitor, bortezomib, potentiates axitinib‐induced cytotoxicity in a p21(Waf/Cip1) dependent manner. Oncotarget 8, 3380–3395. 10.18632/oncotarget.13769 PMC5356889 27926485

[mol212501-bib-0030] Mouawad R , Spano JP , Comperat E , Capron F and Khayat D (2009) Tumoural expression and circulating level of VEGFR‐3 (Flt‐4) in metastatic melanoma patients: correlation with clinical parameters and outcome. Eur J Cancer 45, 1407–1414. 19157860 10.1016/j.ejca.2008.12.015

[mol212501-bib-0031] Muenzner JK , Kunze P , Lindner P , Polaschek S , Menke K , Eckstein M , Geppert CI , Chanvorachote P , Baeuerle T , Hartmann A *et al* (2018) Generation and characterization of hepatocellular carcinoma cell lines with enhanced cancer stem cell potential. J Cell Mol Med 22, 6238–6248. 30280520 10.1111/jcmm.13911 PMC6237557

[mol212501-bib-0032] Murakami T , Toda S , Fujimoto M , Ohtsuki M , Byers HR , Etoh T and Nakagawa H (2001) Constitutive activation of Wnt/β‐catenin signaling pathway in migration‐active melanoma cells: role of LEF‐1 in melanoma with increased metastatic potential. Biochem Biophys Res Comm 288, 8–15. 11594745 10.1006/bbrc.2001.5719

[mol212501-bib-0033] Ostalecki C , Lee JH , Dindorf J , Collenburg L , Schierer S , Simon B , Schliep S , Kremmer E , Schuler G and Baur AS (2017) Multiepitope tissue analysis reveals SPPL3‐mediated ADAM10 activation as a key step in the transformation of melanocytes. Sci Signal 10, eaai8288. 28292959 10.1126/scisignal.aai8288

[mol212501-bib-0034] Pirola L , Ciesielski O and Balcerczyk A (2018) The methylation status of the epigenome: its emerging role in the regulation of tumor angiogenesis and tumor growth, and potential for drug targeting. Cancers 10, 268. 10.3390/cancers10080268 PMC6115976 30103412

[mol212501-bib-0035] Rigamonti N , Kadioglu E , Keklikoglou I , Wyser Rmili C , Leow CC and De Palma M (2014) Role of angiopoietin‐2 in adaptive tumor resistance to VEGF signaling blockade. Cell Rep 8, 696–706. 25088418 10.1016/j.celrep.2014.06.059

[mol212501-bib-0036] Rodier F and Campisi J (2011) Four faces of cellular senescence. J Cell Biol 192, 547–556. 21321098 10.1083/jcb.201009094 PMC3044123

[mol212501-bib-0037] Sfiligoi C , de Luca A , Cascone I , Sorbello V , Fuso L , Ponzone R , Biglia N , Audero E , Arisio R , Bussolino F *et al* (2003) Angiopoietin‐2 expression in breast cancer correlates with lymph node invasion and short survival. Int J Cancer 103, 466–474. 12478661 10.1002/ijc.10851

[mol212501-bib-0038] Shain AH and Bastian BC (2016) From melanocytes to melanomas. Nat Rev Cancer 16, 345–358. 27125352 10.1038/nrc.2016.37

[mol212501-bib-0039] Shao H , Kirkwood JM and Wells A (2015) Tenascin‐C signaling in melanoma. Cell Adh Migr 9, 125–130. 25482624 10.4161/19336918.2014.972781 PMC4422811

[mol212501-bib-0040] Souza CF , Xander P , Monteiro AC , Silva AG , da Silva DC , Mai S , Bernardo V , Lopes JD and Jasiulionis MG (2012) Mining gene expression signature for the detection of pre‐malignant melanocytes and early melanomas with risk for metastasis. PLoS One 7, e44800. 22984562 10.1371/journal.pone.0044800 PMC3439384

[mol212501-bib-0041] Spiric Z , Eri Z and Eric M (2015) Significance of Vascular Endothelial Growth Factor (VEGF)‐C and VEGF‐D in the Progression of Cutaneous Melanoma. Int J Surg Pathol 23, 629–637. 25911567 10.1177/1066896915583694

[mol212501-bib-0042] Streit M and Detmar M (2003) Angiogenesis, lymphangiogenesis, and melanoma metastasis. Oncogene 22, 3172–3179. 12789293 10.1038/sj.onc.1206457

[mol212501-bib-0043] Su J‐L , Chen PS , Chien MH , Chen PB , Chen YH , Lai CC , Hung MC and Kuo ML (2008b) Further evidence for expression and function of the VEGF‐C/VEGFR‐3 axis in cancer cells. Cancer Cell 13, 557–560. 18538739 10.1016/j.ccr.2008.04.021

[mol212501-bib-0044] Su J‐L , Yang PC , Shih JY , Yang CY , Wei LH , Hsieh CY , Chou CH , Jeng YM , Wang MY , Chang KJ *et al* (2008a) The VEGF‐C/Flt‐4 axis promotes invasion and metastasis of cancer cells. Cancer Cell 9, 209–223. 10.1016/j.ccr.2006.02.018 16530705

[mol212501-bib-0045] Tas F , Duranyildiz D , Oguz H , Camlica H , Yasasever V and Topuz E (2006) Circulating serum levels of angiogenic factors and vascular endothelial growth factor receptors 1 and 2 in melanoma patients. Melanoma Res 16, 405–411. 17013089 10.1097/01.cmr.0000222598.27438.82

[mol212501-bib-0046] Thurston G (2003) Role of angiopoietins and tie receptor tyrosine kinases in angiogenesis and lymphangiogenesis. Cell Tissue Res 314, 61–68. 12915980 10.1007/s00441-003-0749-6

[mol212501-bib-0047] Tímár J , Vizkeleti L , Doma V , Barbai T and Rásó E (2016) Genetic progression of malignant melanoma. Cancer Metastasis Rev 35, 93–107. 26970965 10.1007/s10555-016-9613-5

[mol212501-bib-0048] Tsuchida R , Osawa T , Wang F , Nishii R , Das B , Tsuchida S , Muramatsu M , Takahashi T , Inoue T , Wada Y *et al* (2014) BMP4/Thrombospondin‐1 loop paracrinically inhibits tumor angiogenesis and suppresses the growth of solid tumors. Oncogene 33, 3803–3811. 24013228 10.1038/onc.2013.358

[mol212501-bib-0049] Vihinen PP , Hilli J , Vuoristo MS , Syrjänen KJ , Kähäri VM and Pyrhönen SO (2007) Serum VEGF‐C is associated with metastatic site in patients with malignant melanoma. Acta Oncol 46, 678–684. 17562445 10.1080/02841860600965020

[mol212501-bib-0050] Wang CA , Jedlicka P , Patrick AN , Micalizzi DS , Lemmer KC , Deitsch E , Casás‐Selves M , Harrell JC and Ford HL (2012) SIX1 induces lymphangiogenesis and metastasis via upregulation of VEGF‐C in mouse models of breast cancer. J Clin Investig 122, 1895–1906. 22466647 10.1172/JCI59858 PMC3336979

[mol212501-bib-0051] Wei D , Li A , Zhao C , Wang H , Mei C , Khan R and Zan L (2018) Transcriptional regulation by CpG sites methylation in the core promoter region of the bovine SIX1 gene: roles of histone H4 and E2F2. Int J Mol Sci 19, 1–14. 10.3390/ijms19010213 PMC5796162 29337851

[mol212501-bib-0052] Wu W , Ren Z , Liu H , Wang L , Huang R , Chen J , Zhang L , Li P and Xiong Y (2013) Core promoter analysis of porcine Six1 gene and its regulation of the promoter activity by CpG methylation. Gene 529, 238–244. 23954877 10.1016/j.gene.2013.07.102

[mol212501-bib-0053] Zhang X , Fang X , Gao Z , Chen W , Tao F , Cai P , Yuan H , Shu Y , Xu Q , Sun Y *et al* (2014) Axitinib, a selective inhibitor of vascular endothelial growth factor receptor, exerts an anticancer effect in melanoma through promoting antitumor immunity. Anticancer Drugs 25, 204–211. 24135499 10.1097/CAD.0000000000000033

[mol212501-bib-0054] Zhang X and Xu R (2017) Six1 expression is associated with a poor prognosis in patients with glioma. Oncol Lett 13, 1293–1298. 28454249 10.3892/ol.2017.5577 PMC5403215

