# Supplementary material for: Gene expression and promoter methylation of angiogenic and lymphangiogenic factors as prognostic markers in melanoma
Source: Mol Oncol. 2019 May 25;13(6):1433–49. doi: 10.1002/1878-0261.12501 (PMC6547615; doi:10.1002/1878-0261.12501)
Supplement: Supplementary file 1 — Fig. S1. Representative macroscopic images and HE stained tissue sections (4× magnification) of 4C11+ tumors grown on the CAM for 5 days. Fig. S2. Representative macroscopic image and HE staining (4× and 40× magnification) of tumors grown on the CAM. 4C11+ cells were pretreated in vitro with 1 μm of Axitinib for 48 h and applied onto the CAM. Tumors grown were removed after 5 days. Fig. S3. Representative images of VEGFR‐3 and ANGPT2 staining in normal skin and colon, which were used as negative controls of the IHC staining. Table S1. Primers sequences for RT‐qPCR reactions. Table S2. mRNA upregulated in 4C11+ cells in comparison to 4C11− cells as assessed by the NanoString Panel. Table S3. mRNA downregulated in 4C11+ cells in comparison to 4C11− cells as assessed by the NanoString Panel. Table S4. CpGs differentially methylated of Vegfc, Angpt2 and Six1 promoters regions in 4C11+ cells compared to 4C11− cells evaluated by ERRBS. [file MOL2-13-1433-s001.pdf]

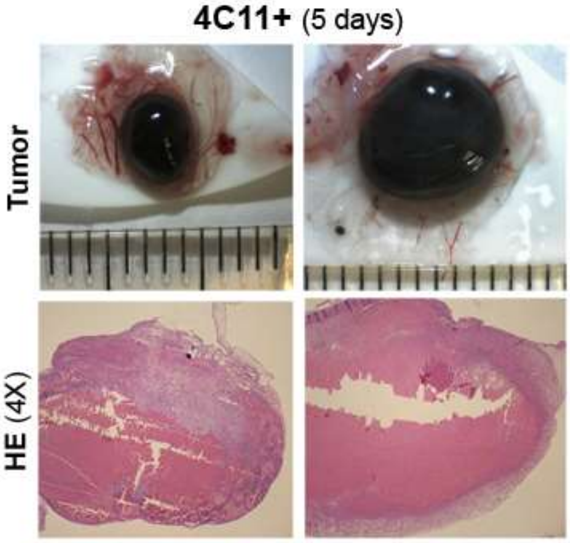

Figure S1. Representative macroscopic images and HE stained tissue sections (4X magnification) of 4C11+ tumors grown on the CAM for 5 days.

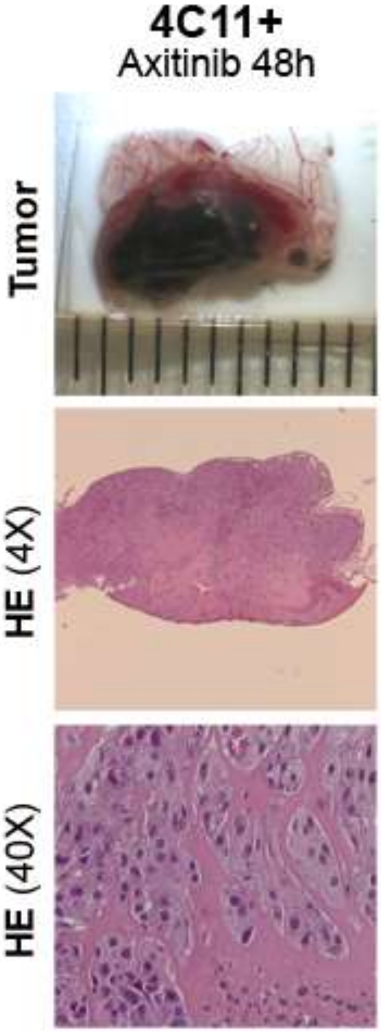

Figure S2. Representative macroscopic image and HE staining (4X and 40X magnification) of tumors grown on the CAM. 4C11+ cells were pre-treated *in vitro* with 1 μM of axitinib for 48 h and applied onto the CAM. Tumors grown were removed after 5 days.

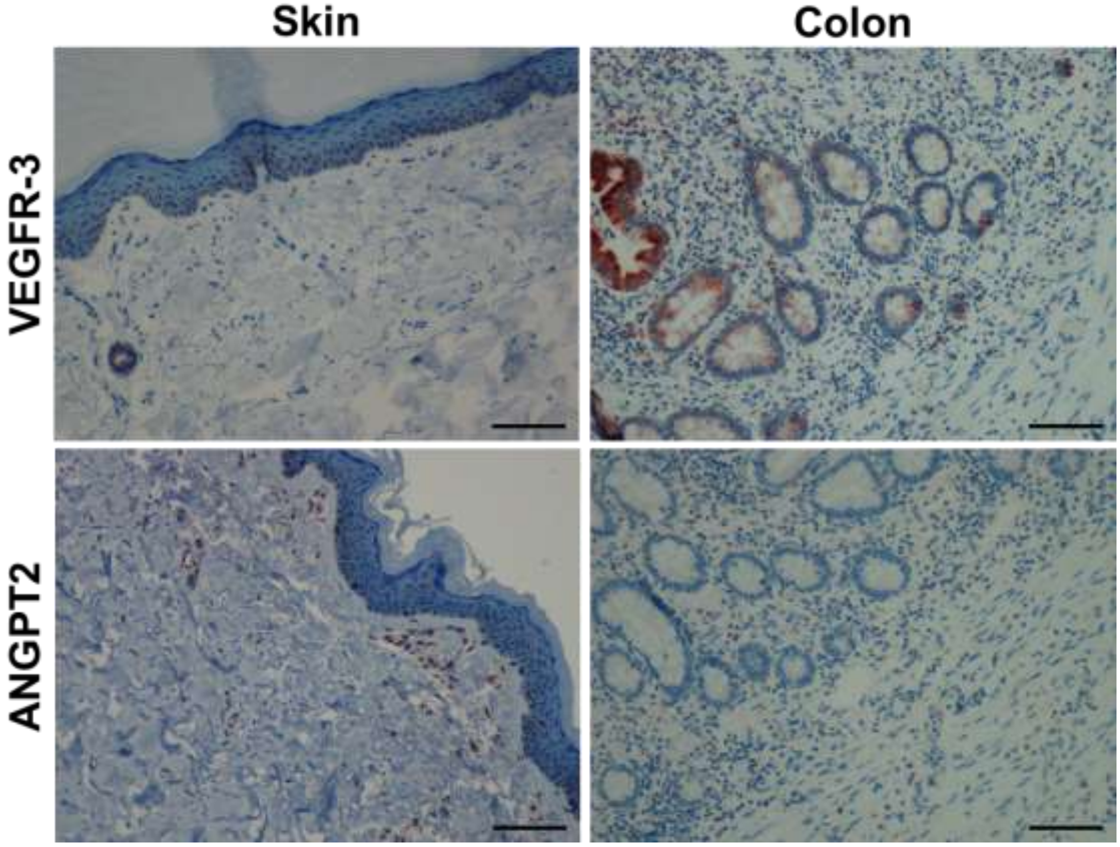

Figure S3. Representative images of VEGFR-3 and ANGPT2 staining in normal skin and colon, which were used as negative controls of the IHC staining. Scale bar: 100 µm.

Table S1. Primers sequences for RT-qPCR reactions

Target	Forward primer sequence (5’-3’)	Reverse primer sequence (5’-3’)
β-actin	ACCGTGAAAAGATGACCCAG	GAGAGCATAGCCCTCGTAGA
Vegfa	GCCTCCGAAACCATGAACTT	GAACTTGATCACTTCATGGGACT
Vegfb	GAATGCAGATCCTCATGATCCA	GTGGTGGGGTATGGCAAC
Vegfc	TGTGCTTCTTGTCTCTGGCG	TGCCTTCAAAAGCCTTGACCTC
Vegfr-1	CTACCTCACCGTGCAAGGAA	TACTTCGGAAGAAGACCGCT
Vegfr-2	CTGCCTACCTCACCTGTTTCC	CTGTCTGTCTGGCTGTCATCT
Vegfr-3	TGGAGGGGAAGAATAAGACGG	GGCTCCGACTCGATACTGAA
Nrp2	GTATCAGATCGTGTTCGAGGGA	TCCTCACCTGCAAAAGCTGA
Angpt2	AGCTCGGTTGCTATCCGTAA	CACAGTAGGCCTTGATCTCCT
Met	CGATCAGCAGTCTGTGCATT	GAGTTTCTCAGCAGGGTCCTA
Six1	GCCAAGGAAAGGGAGAACAC	CCGAGTTCTGGTCTGGACTT

Table S2. mRNAs upregulated in 4C11+ cells in comparison to 4C11- cells as assessed by the NanoString Panel

Gene	Accession #	4C11- counts	4C11+ counts	Fold change (4C11+/4C11-)	Adjusted p-value
Vegfc	NM_009506.2	1.03	738.57	714.31	0.001
Il3ra	NM_008369.1	1.03	431.69	417.51	0.003
Prkaa2	NM_178143.1	1.11	358.84	323.52	0.001
Cntfr	NM_001146080.1	3.33	788.47	236.90	0.022
Col4a5	NM_001163155.1	1.03	193.89	187.52	0.005
Angpt2	NM_007426.3	6.14	879.10	143.15	0.001
Six1	NM_009189.2	1.03	132.52	128.17	0.001
Shc4	NM_199022.2	54.58	3593.57	65.84	0.002
Met	NM_008591.1	57.82	3651.07	63.14	0.002
Id2	NM_010496.3	256.32	15512.01	60.52	0.001
Nr4a3	NM_015743.3	4.08	231.58	56.76	0.029
Eya1	NM_001252192.1	11.63	614.28	52.81	0.007
Ppargc1a	NM_008904.2	29.57	927.61	31.36	0.003
Erbb3	NM_010153.1	25.94	739.74	28.51	0.001
Col1a2	NM_007743.2	8.16	159.15	19.52	0.002
Plcg2	NM_172285.1	13.75	240.87	17.52	0.002
Arnt2	NM_007488.2	7.53	87.62	11.63	0.040
Cdkn1c	NM_009876.3	6.33	56.17	8.87	0.022
Cdkn1a	NM_007669.4	644.58	4679.8	7.26	0.001
Cdk2	NM_016756.4	1837.03	13325.37	7.25	0.016
Col4a4	NM_007735.2	9.11	65.66	7.21	0.005
Pik3cb	NM_029094.3	144.87	1043.35	7.20	0.002
Pik3r3	NM_181585.5	659.70	4741.23	7.19	0.003
Mapk12	NM_013871.3	26.01	185.53	7.13	0.003
Tlr4	NM_021297.2	146.56	953.45	6.51	0.005
Dusp2	NM_010090.2	51.64	311.36	6.03	0.019
Tslp	NM_021367.1	11.93	71.00	5.95	0.006
Map2k6	NM_011943.2	49.97	291.55	5.83	0.001
Rps6ka6	NM_025949.2	35.90	192.74	5.37	0.005
Hmga1	NM_001025427.2	335.54	1731.05	5.16	0.001
Pik3cd	XM_003945690.1	70.90	340.95	4.81	0.010
Cdkn2d	NM_009878.2	139.05	660.23	4.75	0.001
Plcb4	NM_013829.2	215.65	930.08	4.31	0.006
Pla2g3	NM_172791.2	60.90	237.55	3.90	0.010
Efna5	NM_207654.2	404.90	1455.98	3.60	0.004
Gng12	NM_001177559.1	867.06	3105.54	3.58	0.003
Gng7	NM_001038655.1	92.18	314.24	3.41	0.008
Vegfb	NM_011697.2	1406.26	4538.16	3.23	0.001
Prkar2b	NM_011158.3	1156.91	3553.58	3.07	0.015

Mlf1	NM_001039543.2	100.23	307.17	3.06	0.002
Akt1	NM_001165894.1	3068.19	9001.84	2.93	0.005
Bnip3	NM_009760.4	510.87	1460.22	2.86	0.004
Pak3	NM_001195046.1	19.86	56.45	2.84	0.038
Mapk8ip1	NM_001202445.1	478.23	1327.26	2.78	0.003
Mdm2	NM_010786.3	364.70	998.54	2.74	0.001
Il1rap	NM_134103.2	158.03	405.33	2.56	0.005
Ccnd3	NM_007632.2	721.11	1830.27	2.54	0.003
Ccnd2	NM_009829.3	3942.93	9682.12	2.46	0.022
Cdkn2c	NM_007671.2	791.41	1907.82	2.41	0.014
Dusp4	NM_176933.4	2846.68	6821.98	2.40	0.015
Zak	NM_023057.5	1030.00	2466.72	2.39	0.007
Fut8	NM_016893.4	558.50	1303.20	2.33	0.002
Dnmt3a	NM_007872.4	101.88	234.97	2.31	0.013
Pax3	NM_001159520.1	1970.89	4408.51	2.24	0.019
Map2k1	NM_008927.3	1641.88	3645.45	2.22	0.006
Fzd7	NM_008057.3	836.70	1801.63	2.15	0.012
Jag1	NM_013822.2	91.00	187.59	2.06	0.007
Rfc4	NM_145480.1	624.39	1284.15	2.06	0.001
Itga6	NM_008397.3	1532.99	3121.96	2.04	0.010

Table S3. mRNAs downregulated in 4C11+ cells in comparison to 4C11- cells as assessed by the NanoString Panel

Gene	Accession #	4C11- counts	4C11+ counts	Fold change (4C11+/4C11-)	Adjusted p-value
Tnc	NM_011607.1	8956.26	2.43	-3680.76	0.023
Gpc4	NM_008150.2	3956.41	1.41	-2796.48	0.005
Cdkn2a	NM_001040654.1	2814.41	1.01	-2795.53	0.001
Bmp4	NM_007554.2	2384.52	1.01	-2368.52	0.002
Col1a1	NM_007742.3	38729.21	31.72	-1220.91	0.002
Fgfr2	NM_010207.2	1087.60	1.01	-1080.3	0.003
Dusp5	NM_001085390.1	1756.46	1.77	-994.52	0.006
Bmp7	NM_007557.2	1659.76	1.75	-945.8	0.006
Lef1	NM_010703.3	847.56	1.01	-841.87	0.002
Setbp1	NM_053099.2	699.65	1.01	-694.96	0.002
Nog	NM_008711.2	672.90	1.01	-668.39	0.001
Lamc3	NM_011836.3	1270.10	1.91	-666.71	0.018
Pdgfc	NM_019971.2	1944.94	3.03	-642.52	0.021
Cdkn2b	NM_007670.4	610.62	1.01	-606.52	0.002
Tlx1	NM_021901.3	537.56	1.27	-424.44	0.006
Gli1	NM_010296.2	1007.93	2.44	-412.99	0.015
Fgf7	NM_008008.4	400.67	1.01	-397.98	0.007
Wif1	NM_011915.1	3418.44	11.39	-300.18	0.001
Fst	NM_008046.1	293.26	1.04	-282.71	0.001
Cd40	NM_011611.2	262.52	1.01	-260.76	0.003
Col3a1	NM_009930.1	4024.59	16.8	-239.55	0.002
Itga3	NM_013565.2	7493.35	32.64	-229.55	0.001
Egfr	NM_207655.2	215.44	1.01	-213.99	0.004
Pdgfb	NM_011057.3	3998.90	19.25	-207.75	0.001
Fgf9	NM_013518.3	216.34	1.45	-149.27	0.006
Wnt11	NM_001285792.1	403.29	2.76	-146.33	0.020
Mycn	NM_008709.3	134.20	1.01	-133.3	0.004
Hoxa11	NM_010450.2	807.96	6.33	-127.66	0.033
Il6ra	NM_010559.2	1201.88	9.47	-126.98	0.013
Wnt4	NM_009523.1	124.65	1.01	-123.82	0.001
Casp12	NM_009808.4	1366.36	11.26	-121.34	0.010
Il1r1	NM_001123382.1	1793.37	17.18	-104.4	0.001
Etv4	NM_008815.2	857.56	8.69	-98.63	0.032
Gas1	NM_008086.1	3168.54	34.50	-91.83	0.001
Syk	NM_011518.2	153.03	1.72	-88.96	0.028
Wnt7b	NM_009528.2	183.14	2.16	-84.63	0.048
Id1	NM_010495.2	5533.68	65.49	-84.5	0.002
Id4	NM_031166.2	144.58	1.72	-84.16	0.026
Dll1	NM_007865.3	79.08	1.01	-78.55	0.003

Cacna2d1	XM_006535618.1	75.24	1.01	-74.73	0.002
Notch3	NM_008716.2	282.09	3.80	-74.16	0.043
Clcf1	NM_019952.3	72.87	1.01	-72.38	0.002
Epha2	NM_010139.2	518.13	7.16	-72.37	0.010
Gadd45g	NM_011817.1	1924.16	27.52	-69.91	0.002
Cxxc4	NM_001004367.4	111.55	1.72	-64.93	0.033
Flt1	NM_010228.3	64.52	1.01	-64.09	0.006
Igf1	NM_001111274.1	110.83	1.91	-58.18	0.045
Mapk10	NM_001081567.1	521.58	9.00	-57.93	0.024
Ltbp1	NM_019919.2	1321.09	23.33	-56.62	0.001
Lfng	NM_008494.3	2274.35	40.27	-56.48	0.001
Nkd1	NM_027280.3	81.20	1.45	-56.03	0.019
Cacna1g	NM_001112813.2	2583.08	47.58	-54.28	0.001
Pla1a	NM_134102.2	66.01	1.22	-54.15	0.003
Pdgfra	NM_001083316.1	2618.95	49.58	-52.82	0.001
Thbs1	NM_011580.3	2711.07	56.07	-48.36	0.002
Ptch1	NM_008957.2	11480.44	240.9	-47.66	0.001
Col2a1	NM_001113515.2	2177.58	49.37	-44.11	0.003
Flnc	NM_001081185.1	267.85	6.13	-43.66	0.005
Col24a1	NM_027770.2	52.37	1.22	-42.96	0.006
Rin1	NM_145495.2	625.86	15.09	-41.48	0.004
Spp1	NM_009263.3	6469.04	170.07	-38.04	0.001
Zbtb16	NM_001033324.2	54.60	1.53	-35.61	0.008
Col5a1	NM_015734.2	3721.36	110.14	-33.79	0.001
Pdgfrb	NM_008809.1	1231.97	37.23	-33.09	0.002
Fgf17	NM_008004.4	62.13	2.05	-30.31	0.022
Kitl	NM_013598.1	1910.94	67.28	-28.4	0.003
Lif	NM_008501.2	218.53	8.07	-27.07	0.023
Plat	NM_008872.1	884.35	33.52	-26.38	0.017
Wnt6	NM_009526.3	534.15	24.24	-22.03	0.015
Pla2g4a	NM_008869.2	206.59	9.41	-21.96	0.002
Cebpa	NM_007678.3	580.95	26.65	-21.8	0.002
Lama5	NM_001081171.2	2156.30	104.77	-20.58	0.009
Npm2	NM_181345.3	72.85	3.59	-20.31	0.001
Irs1	NM_010570.4	631.92	32.74	-19.3	0.013
Lifr	NM_001113386.1	642.41	34.65	-18.54	0.001
Dtx4	NM_172442.3	456.39	25.48	-17.91	0.006
Efna1	NM_010107.4	1185.56	67.42	-17.58	0.002
Birc3	NM_007464.3	190.80	10.89	-17.51	0.012
Fn1	NM_010233.1	9236.56	531.62	-17.37	0.001
Fzd2	NM_020510.2	1771.57	102.86	-17.22	0.001
Nfatc1	NM_016791.4	520.46	32.46	-16.04	0.012
Igf1r	NM_010513.2	6443.70	410.25	-15.71	0.001
Klf4	NM_010637.3	1255.04	81.43	-15.41	0.013

Pgf	NM_008827.2	850.48	55.92	-15.21	0.001
Fgfr1	NM_001079908.2	2570.86	169.44	-15.17	0.001
Ets2	NM_011809.2	5095.69	345.82	-14.73	0.001
Spry1	NM_011896.2	1278.89	90.86	-14.08	0.008
Fgf5	NM_001277268.1	61.17	4.37	-13.99	0.014
Hes1	NM_008235.2	211.86	15.39	-13.77	0.015
Fas	NM_007987.2	149.99	11.49	-13.06	0.008
Fgfr3	NM_008010.3	1158.70	98.79	-11.73	0.001
Efna3	NM_010108.1	96.78	9.13	-10.6	0.015
Spry4	NM_011898.2	982.64	94.14	-10.44	0.012
Socs3	NM_007707.2	340.57	35.19	-9.68	0.009
Pim1	NM_008842.3	421.87	47.41	-8.9	0.005
Myd88	NM_010851.2	1779.27	207.06	-8.59	0.001
Numbl	NM_010950.2	1171.71	140.35	-8.35	0.001
Cacna1h	NM_021415.4	81.43	10.44	-7.8	0.004
Col27a1	NM_025685.3	170.84	23.82	-7.17	0.002
Camk2b	NM_001174053.1	65.37	9.17	-7.13	0.001
Nfkbiz	NM_030612.1	507.34	73.29	-6.92	0.007
Cacna1c	NM_001159535.1	1351.01	197.92	-6.83	0.005
Arid2	NM_175251.2	205.59	30.56	-6.73	0.007
Pdgfa	NM_008808.3	3624.44	563.99	-6.43	0.001
Tgfb1	NM_011577.1	4589.75	716.79	-6.4	0.005
Hspb1	NM_013560.2	203.48	33.05	-6.16	0.004
Lamc2	NM_008485.3	106.69	17.53	-6.09	0.001
Tcf7l1	NM_001079822.2	2148.28	359.23	-5.98	0.001
Hoxa9	NM_010456.2	316.07	53.02	-5.96	0.010
Efna2	NM_007909.3	155.32	26.88	-5.78	0.004
Mapt	NM_001038609.2	1533.64	265.38	-5.78	0.003
Gadd45b	NM_008655.1	684.72	120.91	-5.66	0.002
Tgfb3	NM_009368.2	2258.00	421.31	-5.36	0.001
Gng2	NM_010315.4	465.32	89.67	-5.19	0.027
Bcl2l1	NM_009743.4	2502.20	503.03	-4.97	0.001
Smad3	NM_016769.3	3945.66	818.86	-4.82	0.004
Notch2	NM_010928.1	1426.56	299.44	-4.76	0.002
Ghr	NM_001048147.1	370.73	79.19	-4.68	0.007
Gadd45a	NM_007836.1	2285.17	490.84	-4.66	0.007
Runx1	NM_001111021.1	1703.10	375.12	-4.54	0.002
Flna	NM_010227.2	3954.78	879.85	-4.49	0.003
Socs1	NM_009896.2	177.31	40.18	-4.41	0.007
Sox9	NM_011448.4	729.97	167.72	-4.35	0.010
Notch1	NM_008714.2	594.15	136.75	-4.34	0.002
Col11a2	NM_009926.1	124.33	28.73	-4.33	0.026
Pbx1	NM_008783.2	1077.28	250.50	-4.3	0.002
H2afx	NM_010436.2	11539.24	2699.03	-4.28	0.004

Casp7	NM_007611.2	1321.99	310.65	-4.26	0.003
Cacnb3	NM_007581.2	2080.29	489.38	-4.25	0.002
Cdk6	NM_009873.2	1151.40	276.80	-4.16	0.002
Fancb	NM_001146081.1	534.78	129.46	-4.13	0.002
Amer1	NM_175179.4	880.35	219.65	-4.01	0.003
Fos	NM_010234.2	361.97	92.20	-3.93	0.03
Erbb2	NM_001003817.1	1478.12	381.72	-3.87	0.007
Dvl1	NM_010091.3	1616.84	430.6	-3.75	0.004
Map3k14	NM_016896.3	242.67	65.51	-3.7	0.001
Kmt2d	NM_001033276.1	5440.87	1469.42	-3.7	0.008
Rac3	NM_133223.4	328.72	89.42	-3.68	0.024
Lepr	NM_010704.2	55.92	15.43	-3.62	0.032
Ddb2	NM_028119.5	215.90	60.17	-3.59	0.003
Pla2g4c	NM_001168504.1	121.21	34.06	-3.56	0.025
Nthl1	NM_008743.2	776.61	219.69	-3.54	0.015
Nsd1	NM_008739.3	991.29	282.49	-3.51	0.006
Sfn	NM_018754.2	164.84	47.44	-3.47	0.007
Prkca	NM_011101.3	708.48	212.42	-3.34	0.005
Dusp8	NM_008748.1	167.36	51.04	-3.28	0.009
Tiam1	NM_009384.2	1009.77	309.31	-3.26	0.004
Nf1	NM_010897.2	3043.32	939.49	-3.24	0.002
Mapk8	NM_016700.3	2287.61	713.20	-3.21	0.001
Fance	NM_001163819.1	288.61	91.26	-3.16	0.024
Med12	NM_021521.2	3201.69	1027.01	-3.12	0.007
Kdm5c	NM_013668.3	3147.53	1017.10	-3.09	0.004
Cbl	NM_007619.2	6449.60	2090.48	-3.09	0.001
Stat3	NM_213659.2	3620.35	1191.54	-3.04	0.022
Bcor	NM_029510.3	1213.46	402.82	-3.01	0.005
Hdac10	NM_199198.1	451.62	150.84	-2.99	0.011
Dusp10	NM_022019.5	363.38	123.24	-2.95	0.002
Insr	NM_010568.2	591.81	200.44	-2.95	0.024
Ep300	NM_177821.6	5931.57	2128.35	-2.79	0.002
Smad1	NM_008539.3	1149.92	415.92	-2.76	0.002
Fzd3	NM_021458.1	1528.05	553.69	-2.76	0.002
Whsc1l1	NM_001001735.1	4134.58	1504.76	-2.75	0.003
Jun	NM_010591.2	1658.64	612.66	-2.71	0.007
Hdac5	NM_010412.3	2248.61	842.63	-2.67	0.005
Socs2	NM_001168655.1	283.43	106.71	-2.66	0.032
Trp53	NM_011640.1	3010.44	1135.96	-2.65	0.001
Elk1	NM_007922.4	122.09	46.50	-2.63	0.013
Acvr1b	NM_007395.3	1215.76	471.77	-2.58	0.011
Axin1	NM_001159598.1	1206.87	479.27	-2.52	0.001
Smc1a	NM_019710.2	3988.19	1588.19	-2.51	0.001
Asxl1	NM_001039939.1	3447.26	1399.54	-2.46	0.004

Setd2	NM_001081340.2	3830.51	1557.25	-2.46	0.004
Rps6ka5	NM_153587.2	206.43	84.40	-2.45	0.027
Mutyh	NM_001159581.1	210.44	88.22	-2.39	0.043
Tfdp1	NM_009361.2	15147.30	6359.97	-2.38	0.002
Ccnd1	NM_007631.1	7435.60	3136.18	-2.37	0.007
Suv39h2	NM_022724.3	1275.51	544.62	-2.34	0.004
Fancc	NM_007985.2	436.71	188.36	-2.32	0.007
Map3k1	NM_011945.2	1184.37	511.98	-2.31	0.007
Skp2	NM_013787.2	2131.62	922.72	-2.31	0.007
Zic2	NM_009574.3	439.74	191.91	-2.29	0.001
Axin2	NM_015732.4	320.2	141.94	-2.26	0.015
Hspa2	NM_008301.4	233.51	104.18	-2.24	0.021
Alkbh2	NM_175016.2	392.06	176.27	-2.22	0.008
Hdac4	NM_207225.1	755.18	340.63	-2.22	0.030
Cdc7	NM_001271566.1	753.07	345.26	-2.18	0.002
Bcl2	NM_009741.3	724.38	339.71	-2.13	0.005
Tcf3	NM_001164147.1	93.92	44.77	-2.1	0.022
Kmt2c	NM_001081383.1	565.27	268.92	-2.1	0.031
Bid	NM_007544.3	1212.45	583.41	-2.08	0.001
Daxx	NM_007829.3	426.89	206.53	-2.07	0.007
Ikbkg	NM_178590.2	791.21	387.72	-2.04	0.012
Pik3r2	NM_008841.2	2448.84	1208.75	-2.03	0.019
Casp3	NM_009810.2	1849.59	913.74	-2.02	0.004
Pkmyt1	NM_023058.3	980.34	490.05	-2,00	0.054

Table S4. CpGs differentially methylated of *Vegfc*, *Angpt2* and *Six1* promoters regions in 4C11+ cells compared to 4C11- cells evaluated by ERRBS.

Gene symbol	Chromossome	CpG location	CpG distance to TSS*	Methylation difference (%)	Adjusted p-value
Vegfc	chr8	54077678	147	-29.51	0.006
Vegfc	chr8	54077737	206	-40.69	< 0.001
Vegfc	chr8	54077747	216	-31.85	0.006
Vegfc	chr8	54077752	221	-30.39	0.007
Angpt2	chr8	18740192	-1370	-85.37	0.001
Angpt2	chr8	18740302	-1260	-74.95	< 0.001
Angpt2	chr8	18740305	-1257	-89.34	< 0.001
Angpt2	chr8	18740342	-1220	-90.73	0.006
Angpt2	chr8	18740345	-1217	-96.15	< 0.001
Angpt2	chr8	18740346	-1216	-93.87	0.002
Angpt2	chr8	18740348	-1214	-94.01	0.002
Angpt2	chr8	18740355	-1207	-91.35	< 0.001
Angpt2	chr8	18740372	-1190	-88.67	< 0.001
Angpt2	chr8	18740410	-1152	-83.51	< 0.001
Angpt2	chr8	18740433	-1129	-92.41	< 0.001
Six1	chr12	73045213	-1499	-82.61	< 0.001
Six1	chr12	73045231	-1481	-73.68	0.005
Six1	chr12	73045233	-1479	-79.34	0.004
Six1	chr12	73045240	-1472	-67.18	< 0.001
Six1	chr12	73045253	-1459	-84.23	< 0.001
Six1	chr12	73045254	-1458	-81.10	0.004
Six1	chr12	73045285	-1427	-75.35	< 0.001
Six1	chr12	73045352	-1360	-08.25	0.001
Six1	chr12	73045354	-1358	-79.60	0.002
Six1	chr12	73045388	-1324	-82.99	< 0.001
Six1	chr12	73045408	-1304	-78.28	< 0.001
Six1	chr12	73045425	-1287	-77.94	< 0.001
Six1	chr12	73045429	-1283	-85.25	< 0.001
Six1	chr12	73045433	-1279	-84.61	< 0.001
Six1	chr12	73045454	-1258	-86.84	< 0.001
Six1	chr12	73045529	-1183	-06.63	< 0.001
Six1	chr12	73045571	-1141	-83.56	0.005
Six1	chr12	73045574	-1138	-88.32	< 0.001
Six1	chr12	73045584	-1128	-76.74	< 0.001
Six1	chr12	73045607	-1105	-70.86	0.009
Six1	chr12	73045610	-1102	-77.78	< 0.001
Six1	chr12	73045614	-1098	-74.78	0.006
Six1	chr12	73045723	-989	-81.04	< 0.001
Six1	chr12	73045726	-986	-86.59	0.001

Six1	chr12	73045729	-983	-82.08	< 0.001
Six1	chr12	73045735	-977	-82.16	< 0.001
Six1	chr12	73045760	-952	-81.90	< 0.001
Six1	chr12	73045767	-945	-81.25	< 0.001
Six1	chr12	73045770	-942	-81.58	< 0.001
Six1	chr12	73045788	-924	-80.95	< 0.001
Six1	chr12	73045789	-923	-74.96	0.001
Six1	chr12	73045791	-921	-79.33	0.001
Six1	chr12	73045792	-920	-77.17	< 0.001
Six1	chr12	73045797	-915	-82.02	< 0.001
Six1	chr12	73045798	-914	-77.25	< 0.001
Six1	chr12	73045815	-897	-80.80	< 0.001
Six1	chr12	73045831	-881	-86.68	0.002
Six1	chr12	73045832	-880	-77.63	0.001
Six1	chr12	73045841	-871	-80.66	0.004
Six1	chr12	73045842	-870	-76.44	0.004
Six1	chr12	73045857	-855	-83.02	0.004
Six1	chr12	73045858	-854	-77.42	0.003
Six1	chr12	73045860	-852	-88.13	0.003
Six1	chr12	73045861	-851	-76.95	0.004
Six1	chr12	73045862	-850	-85.92	0.003
Six1	chr12	73045863	-849	-07.40	0.002
Six1	chr12	73045881	-831	-79.99	0.001
Six1	chr12	73045884	-828	-76.38	< 0.001
Six1	chr12	73045889	-823	-76.61	0.002
Six1	chr12	73045903	-809	-72.10	0.007
Six1	chr12	73046703	-9	-63.20	< 0.001
Six1	chr12	73046728	16	-84.18	< 0.001
Six1	chr12	73046733	21	-84.24	0.001
Six1	chr12	73046744	32	-86.96	0.002
Six1	chr12	73046746	34	-81.40	0.001
Six1	chr12	73046751	39	-78.89	0.009
Six1	chr12	73046776	64	-81.89	< 0.001
Six1	chr12	73046780	68	-87.25	< 0.001
Six1	chr12	73046783	71	-68.37	0.002
Six1	chr12	73046791	79	-82.48	< 0.001
Six1	chr12	73046808	96	-82.94	< 0.001
Six1	chr12	73046810	98	-86.47	< 0.001
Six1	chr12	73046813	101	-86.64	< 0.001
Six1	chr12	73046817	105	-86.62	< 0.001
Six1	chr12	73046821	109	-87.02	< 0.001
Six1	chr12	73046833	121	-84.27	0.002
Six1	chr12	73046835	123	-81.52	< 0.001
Six1	chr12	73046846	134	-08.50	< 0.001

Six1	chr12	73046851	139	-88.72	< 0.001
Six1	chr12	73046853	141	-90.43	< 0.001
Six1	chr12	73046864	152	-82.56	< 0.001
Six1	chr12	73046915	203	-90.43	< 0.001

*TSS – Transcription start site
